# Arctic cyanobacterial mat community diversity decreases with latitude across the Canadian Arctic

**DOI:** 10.1093/femsec/fiae067

**Published:** 2024-04-23

**Authors:** Patrick M Hooper, David Bass, Edward J Feil, Warwick F Vincent, Connie Lovejoy, Christopher J Owen, Stephania L Tsola, Anne D Jungblut

**Affiliations:** Science Department, Natural History Museum, Cromwell Road, London, SW7 5BD, United Kingdom; Milner Centre for Evolution, Department of Life Sciences, University of Bath, Claverton Down, Bath, BA2 7AY, United Kingdom; Science Department, Natural History Museum, Cromwell Road, London, SW7 5BD, United Kingdom; Centre for Environment, Fisheries and Aquaculture Science (Cefas), Weymouth Laboratory, Barrack Road, Weymouth, DT4 8UB, United Kingdom; Centre for Sustainable Aquaculture Futures, University of Exeter, Stocker Road, Exeter, EX4 4QD, United Kingdom; Milner Centre for Evolution, Department of Life Sciences, University of Bath, Claverton Down, Bath, BA2 7AY, United Kingdom; Département de Biologie, Takuvik International Research Laboratory and Institut de Biologie Intégrative et des Systèmes, Université Laval, Québec, QC, G1V 0A6, Canada; Centre d’études nordiques (CEN), Université Laval, Québec, QC, G1V 0A6, Canada; Département de Biologie, Takuvik International Research Laboratory and Institut de Biologie Intégrative et des Systèmes, Université Laval, Québec, QC, G1V 0A6, Canada; Centre d’études nordiques (CEN), Université Laval, Québec, QC, G1V 0A6, Canada; Québec Océan, Université Laval, Québec, QC, G1V 0A6, Canada; UCL Genetics Institute, University College London, Gower Street, London, WC1E 6BT, United Kingdom; School of Biological and Behavioural Sciences, Queen Mary University of London, Mile End Road, London, E1 4NS, United Kingdom; Science Department, Natural History Museum, Cromwell Road, London, SW7 5BD, United Kingdom

**Keywords:** 16S rRNA, 18S rRNA, aquatic ecosystems, Arctic, microbial mats, protists

## Abstract

Cyanobacterial mats are commonly reported as hotspots of microbial diversity across polar environments. These thick, multilayered microbial communities provide a refuge from extreme environmental conditions, with many species able to grow and coexist despite the low allochthonous nutrient inputs. The visibly dominant phototrophic biomass is dependent on internal nutrient recycling by heterotrophic organisms within the mats; however, the specific contribution of heterotrophic protists remains little explored. In this study, mat community diversity was examined along a latitudinal gradient (55–83°N), spanning subarctic taiga, tundra, polar desert, and the High Arctic ice shelves. The prokaryotic and eukaryotic communities were targeted, respectively, by V4 16S ribosomal RNA (rRNA) and V9 18S rRNA gene amplicon high-throughput sequencing. Prokaryotic and eukaryotic richness decreased, in tandem with decreasing temperatures and shorter seasons of light availability, from the subarctic to the High Arctic. Taxonomy-based annotation of the protist community revealed diverse phototrophic, mixotrophic, and heterotrophic genera in all mat communities, with fewer parasitic taxa in High Arctic communities. Co-occurrence network analysis identified greater heterogeneity in eukaryotic than prokaryotic community structure among cyanobacterial mats across the Canadian Arctic. Our findings highlight the sensitivity of microbial eukaryotes to environmental gradients across northern high latitudes.

## Teaser

Microbial diversity in freshwater microbial mats declines with decreasing temperature and light availability, from the subarctic to High Arctic, highlighting a sensitivity to environmental gradients and climate change.

## Introduction

The Arctic is warming considerably faster than the global average, altering aquatic environments and the biodiversity within (Rantanen et al. 2022). Microbial mats dominated by phototrophic oscillatorian cyanobacteria, hereafter referred to as cyanobacterial mats, are a common feature of aquatic ecosystems in the polar regions (Vincent et al. 2000, Jungblut et al. 2010). Cyanobacterial mats are often the major drivers of organic carbon fixation and nutrient cycling in Arctic aquatic ecosystems (Vincent et al. 2000, Varin et al. 2010). The multilayered three-dimensional structure of cyanobacterial mats creates a microhabitat for diverse microbial communities, providing refuge from the stresses imposed by polar environments, including persistent low temperatures, low allochthonous nutrient input, and repeated extreme annual light and freeze–thaw cycles (Vincent et al. 2000, Jungblut et al. 2012, Velázquez et al. 2017). Internal nutrient recycling and scavenging by heterotrophic bacteria, fungi, and viruses in the cyanobacterial mat communities creates a nutrient-rich environment within often oligotrophic Arctic aquatic ecosystems (Varin et al. 2010, Vigneron et al. 2018).

Eukaryotic microbes and microfauna have been observed in polar microbial mat communities since the earliest microscopic investigation of Antarctic mats (Murray 1910). More recently, 18S ribosomal RNA (18S rRNA) gene clone library surveys (Jungblut et al. 2012) and metagenomic (Varin et al. 2010, Vigneron et al. 2018) sequencing of microbial mat communities in the Canadian High Arctic identified algae and heterotrophic protists, microfauna, and fungi. Almela et al. (2019) demonstrated using DNA sequencing and stable isotope assays that multiple trophic levels are present within Antarctic microbial mat communities, where carbon is transferred from primary production by cyanobacteria and diatoms to metazoan consumers, including rotifers, tardigrades, and nematodes, and recycled by fungal decomposers. However, their approach did not consider heterotrophic protists. Heterotrophic protists exhibit diverse ecological functions, including bacterivores, eukaryvores, mixotrophs, parasites, and pathogens, meaning that protists commonly occupy multiple trophic levels in environmental microbiomes (Arndt and Nomdedeu 2016). Moreover, protist grazers contribute to nutrient cycling in temperate stream biofilm communities (Battin et al. 2016). A comprehensive analysis of the eukaryotic community is therefore required to obtain a more complete understanding of the potential trophic interactions and contribution to nutrient recycling by microbial eukaryotes within polar cyanobacterial mat communities (Jungblut et al. 2012).

The Canadian North spans distinct ecozones across a continuous gradient of environmental severity from the subarctic boreal taiga, through the open tundra, to the polar desert and ice shelves of the High Arctic, each characterized by distinct vegetation and climatic regimes, but all consistently rich in aquatic ecosystems (Vincent and Laybourn-Parry 2008, Wrona et al. 2016). Streams, rivers, and meltwater ponds are consistent across the Arctic region, while perennially ice-covered lakes and ice shelf meltwater ponds are specific to the High Arctic, due to the extended periods of below freezing temperatures (Vincent et al. 2000, Jungblut et al. 2017). While the habitat characteristics of Arctic aquatic ecosystems vary broadly, they all commonly host benthic cyanobacterial mat communities (Jungblut et al. 2010). Accordingly, cyanobacterial mats are useful as comparative microbial communities for diverse biogeographical questions and for elucidating the influence of abiotic drivers, including potential climate-driven environmental change, on microbial diversity.

To date, most studies on cyanobacterial mats in the Arctic region have been restricted to a few specific habitats or have been limited to either prokaryotes or microbial eukaryotes (de los Ríos et al. 2004, Varin et al. 2012, Jungblut et al. 2017). In this study, we evaluated the prokaryotic and eukaryotic diversity in cyanobacterial mats in terrestrial aquatic environments of the Canadian North, across a broad latitudinal gradient from the subarctic taiga to the High Arctic ice shelves (55–83°N), using 16S rRNA and 18S rRNA gene amplicon high-throughput sequencing. We hypothesized that prokaryotic and eukaryotic diversity within cyanobacterial mat communities would decrease with increasing latitude across the Canadian Arctic, in response to gradients of less favourable climatic conditions for net growth, similar to terrestrial fauna and flora (Wrona et al. 2016). The influence of environmental variables on community richness and evenness was assessed. The functional contribution of the protist community was further assessed following taxonomy-based assignment into broad trophic functions (Adl et al. 2019, Singer et al. 2021). Finally, co-occurrence networks were created to identify interactions within and between the prokaryotic and eukaryotic communities, within the cyanobacterial mats.

## Materials and methods

### Sample collection and environmental conditions

Cyanobacterial mats were collected from terrestrial aquatic environments across the Canadian North. Benthic mat samples from lakes and ponds were collected near the hamlets of Kuujjuarapik, Umiujaq, Cambridge Bay, and Resolute, and from Bylot Island, Ward Hunt Lake on Ward Hunt Island, and Antoniades Pond on Ellesmere Island. Less terrestrially influenced mats were collected from ice-based surface meltwater ponds from the Markham Ice Shelf and Ward Hunt Ice Shelf (Supplementary Fig. S1; Table 1). Mat samples were collected in triplicate, except for Bylot Island and Resolute sites due to sampling time constraints. Samples were collected at water depths of 10–20 cm using a sterilized steel spatula and transferred to a sterile Falcon™ 50-ml centrifuge tube. Samples were stored at −20°C in field laboratories before being transported to the Natural History Museum, London, UK, for storage at −80°C until DNA extraction. At each sample site, GPS coordinates, water specific conductivity (µS cm^−1^), pH, and temperature were measured at the sampling depth using a pH/Con 10 series probe (Oakton Instruments, UK). Average monthly air temperature data were extracted from Nordicana D (CEN 2020, 2021, 2022a,b) and the Environment and Climate Change Canada Historical Climate Data (McKenney et al. 2011, MacDonald et al. 2020) archives.

**Table 1.** Sampling sites for mat communities and measured water conditions in the Canadian Arctic.

Sample ID	Sampling region	Water body	Sampling period	Coordinates (°)	Temperature (°C)	pH	Conductivity (µS cm^−1^)
KJ1	Kuujjuarapik	Pond	August 2017	55.283, −77.739	19.63	7.27	60.33
KJ2	Kuujjuarapik	Lake	August 2017	55.287, −77.737	18.97	7.95	48.00
KJ3	Kuujjuarapik	Pond	August 2017	55.314, −77.735	20.83	7.48	65.67
KJ4	Kuujjuarapik	Pond	August 2017	55.323, −77.715	17.33	7.16	50.67
KJ5	Kuujjuarapik	Pond	August 2017	55.326, −77.717	17.00	6.70	38.00
KJ6	Kuujjuarapik	Pond	August 2017	55.33, −77.7	19.60	9.01	70.67
KJ7	Kuujjuarapik	Pond	August 2017	55.334, −77.697	16.57	7.72	109.00
KJ8	Kuujjuarapik	Pond	August 2017	55.361, −77.651	14.80	8.83	263.33
UM1	Umiujaq	Lake inflow	August 2009	56.155, −76.308	17.97	6.93	36.90
UM2	Umiujaq	Pond	August 2009	56.62, −76.626	17.57	7.06	57.53
UM3	Umiujaq	Pond	August 2009	56.622, −76.601	17.77	7.37	36.57
UM4	Umiujaq	Pond	August 2009	56.682, −76.707	16.20	5.90	29.80
UM5	Umiujaq	Rock pool	August 2009	56.709, −76.616	15.50	9.66	112.50
UM6	Umiujaq	Pond	August 2009	56.789, −76.296	15.00	6.89	36.63
UM7	Umiujaq	Pond	August 2009	56.792, −76.313	15.57	6.88	29.77
UM8	Umiujaq	Pond	August 2009	56.795, −76.517	16.80	6.37	32.13
CB1	Cambridge Bay	Pond	August 2017	69.129, −105.087	17.10	9.36	759.33
CB2	Cambridge Bay	Lake	August 2017	69.136, −105.053	8.83	8.85	375.33
CB3	Cambridge Bay	Pond	August 2017	69.184, −104.691	10.20	9.03	991.67
CB4	Cambridge Bay	Pond	August 2017	69.184, −104.696	10.07	9.20	764.33
CB5	Cambridge Bay	Lake	August 2017	69.21, −104.787	17.27	8.23	1302.67
CB6	Cambridge Bay	Lake	August 2017	69.243, −104.76	17.57	8.37	302.00
CB7	Cambridge Bay	Pond	August 2017	69.243, −104.76	11.90	8.88	1363.67
CB8	Cambridge Bay	Pond	August 2017	69.266, −104.754	15.37	8.45	551.00
BY1	Bylot Island	Pond	July 2009	73.15, −79.967	13.30	7.55	100.00
BY2	Bylot Island	Pond	July 2009	73.15, −79.967	15.30	6.59	79.00
RE1	Resolute	Pond	August 2008	74.763, −95.212	NA	8.00	560.00
RE2	Resolute	Pond	August 2008	74.79, −95.09	6.80	7.34	539.00
WH	Ward Hunt Lake, Ward Hunt Island	Lake	July 2007	83.167, −74.345	1.83	7.87	113.30
AP	Antoniades Pond, Ellesmere Island	Pond	July 2007	83.233, −75.445	6.00	8.28	137.00
MKIS1	Markham Ice Shelf	Ice shelf meltwater pond	July 2007	83.266, −71.726	1.10	6.52	492.00
MKIS2	Markham Ice Shelf	Ice shelf meltwater pond	July 2007	83.266, −71.726	2.80	6.79	779.00
MKIS3	Markham Ice Shelf	Ice shelf meltwater pond	July 2007	83.266, −71.749	1.40	6.29	640.00
WHIS1	Ward Hunt Ice Shelf	Ice shelf meltwater pond	July 2007	83.33, −74.511	0.80	6.98	364.00
WHIS2	Ward Hunt Ice Shelf	Ice shelf meltwater pond	July 2007	83.331, −74.496	1.50	6.24	740.00
WHIS3	Ward Hunt Ice Shelf	Ice shelf meltwater pond	July 2007	83.331, −74.451	0.40	6.14	50.30

### DNA extraction, polymerase chain reaction, and amplicon sequencing

DNA was extracted from cyanobacterial mat samples using the PowerBiofilm™ DNA Extraction Kit (MO BIO Laboratories, Carlsbad, CA, USA), following the manufacturer’s guidelines. In the absence of triplicate samples, triplicate DNA extractions were carried out on portions of the same mat sample from Resolute and Bylot Island. Extracted DNA was amplified by polymerase chain reaction (PCR). For 16S rRNA gene sequencing, 260 bp of the V4 region of the prokaryotic 16S rRNA gene was amplified using the 515f-806r primers (Caporaso et al. 2012). For 18S rRNA gene sequencing, 130 bp of the V9 region of the eukaryotic 18S rRNA gene was amplified using 1391f-EukBr primers (Amaral-Zettler et al. 2009). For each sample, triplicate PCR samples were amplified with 0.5, 1, and 1.5 µl volumes of template DNA, to accommodate potential amplification bias. For sequencing, the primers were composed of an Illumina adaptor, unique Golay barcode (reverse primers only), primer pad, and primer linker sequences. PCRs were carried out in 20 µl reaction volumes consisting of 4 µl 5x GoTaq reaction buffer (Promega, Madison, WI, USA), 2 µl MgCl (25 mM), 0.8 µl bovine serum albumin (20 mg ml^−1^), 0.16 µl dNTPs (25 mM), 1 µl forward primer (10 µM), 1 µl reverse primer (10 µM), 9.84 µl PCR-grade deionized H_2_O, and 0.2 µl GoTaq® DNA polymerase (Promega, Madison, WI, USA). The thermocycling conditions consisted of an initial denaturation at 94°C for 4 min, followed by 35 cycles of denaturation at 94°C for 30 s, annealing at 50°C for 30 s, extension at 72°C for 2 min, and a final extension at 72°C for 7 min. PCR products were visualized by gel electrophoresis, run on a 1% agarose gel for 35 min at 100 V. PCR products were purified using the AxyPrep™ Clean-Up Protocol (Axygen Scientific, Corning, NY, USA), following the manufacturer’s guidelines. Purified triplicate PCR products were pooled and final concentrations were determined in duplicate using a Qubit 2.0 Fluorometer (ThermoFisher Scientific, Waltham, MA, USA). The amplicons were combined at equimolar concentrations prior to 2 × 250 bp sequencing on the MiSeq™ System (Illumina, San Diego, CA, USA) at the Natural History Museum, London, UK. Samples were demultiplexed based on the unique Golay barcodes per sample. The raw sequences were submitted to GenBank SRA (BioProject ID: PRJNA954357).

### Bioinformatic analyses

Sequenced and demultiplexed forward and reverse DNA reads were processed and filtered using the DADA2 pipeline in RStudio (*dada2* v1.20.0, Callahan et al. 2016; R v4.1.0, R Core Team 2021; RStudio v1.4.1717, RStudio Team 2020). The V4 16S rRNA gene reads were taxonomically assigned using the SILVA database v138.1 (Quast et al. 2013, Yilmaz et al. 2014) and the PR2 database v4.13.0 (Guillou et al. 2012) for the V9 18S rRNA gene reads.

Amplicon sequence variant (ASV) sequence tables, taxonomic assignments, and sample metadata were combined in the *phyloseq* v1.38.0 package (McMurdie and Holmes 2013). The 16S rRNA gene and 18S rRNA gene ASV tables were filtered after taxonomic assignment. Initially, four samples were removed from the 18S rRNA gene sequencing run as they contained fewer than five ASVs (CB8.3, CB1.2, BY2.2, and MKIS2). A raw sequence table of 38 631 unique ASVs was produced from the 16S rRNA gene reads. Prevalence filtering removed all ASVs identified in a single sample from the 16S rRNA gene read sequence table, leaving 19 481 unique ASVs. Furthermore, ASVs assigned to eukaryotic taxa were removed, along with any mitochondria or chloroplast sequences, and sequences without phylum-level assignment, leaving 17 952 unique ASVs in the 16S rRNA gene sequence table. For the 18S rRNA gene, a raw sequence table of 11 920 unique ASVs was produced from the reads. Prevalence filtering was not performed due to the expected lower abundance of eukaryotic microorganisms in microbial mat communities (Jungblut et al. 2012). Taxonomic filtering removed all non-eukaryotic reads and any sequences not assigned at the supergroup level. The eukaryotic phyla Picozoa, Telonemia, and Stramenopiles_X were also removed as they were extremely rare, having only single ASVs present in single samples. Phylogenetic distances were then used to agglomerate proximally close ASVs using the ‘*tip_glom*’ function in *phyloseq* (*H* = 0.01). Phylogenetic trees were produced from the filtered 16S rRNA and 18S rRNA gene ASVs using the *DECIPHER* v2.22.0 package (Wright 2020). Agglomeration produced a final ASV dataset of 15 250 unique ASVs from the 16S reads and 9354 unique ASVs from the 18S reads.

### Statistical analyses

Statistical analyses were conducted using the *phyloseq* v1.38.0 (McMurdie and Holmes 2013) and *vegan* v2.5.7 (Oksanen et al. 2020) packages. ASV count data were not rarefied prior to diversity estimates to avoid inflation of false positives (McMurdie and Holmes 2014). Alpha-diversity measures were calculated for each sample individually. Changes in richness (ACE diversity index) and evenness (Shannon and inverse Simpson diversity indices) with latitude were assessed by linear regression. Differences in alpha diversity between sampling sites were tested by nonparametric Kruskal–Wallis and Wilcoxon tests. For all further statistical and taxonomic analyses, the ASV count data from the replicate samples from each water body were averaged. Several functions in the *vegan* v2.5.7 package (Oksanen et al. 2020) were used for statistical analyses. The ‘*cca*’ function was used to produce canonical correspondence analysis (CCA) plots to identify associations with measured environmental variables. Associations between sample composition and water temperature, pH, and conductivity, average air temperature, and geographic distance were assessed using the ‘*mantel*’ function. Bray–Curtis dissimilarity matrixes were calculated on the averaged relative-abundance transformed data for each water body. Nonmetric multidimensional scaling (NMDS) of the 16S rRNA and 18S rRNA gene communities and significant differences in composition between sampling sites were assessed using the ‘*ANOSIM*’ function with 999 permutations.

### Functional annotation of 18S rRNA gene ASVs

Protist ASVs were assigned to trophic functional groups following taxonomic assignment. Protists were classified as autotrophic phototrophs and mixotrophs, heterotrophic bacterivores, eukaryvores and omnivores, pathogens, or parasites. The Cercozoa (Dumack et al. 2019), Chrysophyceae (Bock et al. 2022), Ciliophora, and Dinoflagellata (Adl et al. 2019) were primarily assigned based on published taxonomic summaries for the respective groups. Within PR2, Chrysophyceae are grouped into phylogenetic clades (Guillou et al. 2012). For this study, these clades were assigned a trophic function based on comparison to the taxonomic functional database by Bock et al. (2022). ASVs unassigned at the family level in clades B1, B2, and E were defined as mixotrophic—as all taxonomic lineages in these clades were assigned as such. Clades C, D, and F contain a mix of heterotrophic and mixotrophic lineages and were therefore not assigned a function but can be assumed to have phagotrophic capability (Bock et al. 2022).

### Co-occurrence network analyses of 16S rRNA and 18S rRNA gene ASVs

Co-occurrence networks were constructed using the *CoNet* v1.1.1 plugin (Faust and Raes 2016) in *Cytoscape* v3.9.1 (Shannon et al. 2003). For the prokaryotic protist interaction network, metazoa and embryophyte-assigned ASVs were removed from the 18S rRNA gene reads. The 16S rRNA and 18S rRNA gene ASVs were relative abundance transformed, to correct for differential sequencing depth. To reduce false positives, ASVs were filtered to a minimum mean relative abundance of 1 × 10^−5^ and a minimum prevalence of 25% (8/32 samples). For the eukaryotic protist interaction network, metazoa and embryophyte ASVs were retained, and samples were filtered to the same abundance and prevalence thresholds. Copresence (positive) and mutual exclusion (negative) associations were inferred by five separate methods: Spearman’s rank correlation and Kendall correlation, chosen as they do not assume linearity; mutual information (distance between probability distributions); Bray–Curtis; and Kullback–Leibler distance dissimilarity methods. To minimize sparsity effects, ASV rows with more than or equal to five null (0) values were removed (*row_minocc* = 5). All filtered rows were summed into a single row that was kept for further processing. Samples were then standardized by conversion to column-wise proportional abundances (*col_norm*). The initial network was created using an automatic threshold of 1000 positive edges by all four measures. For each measure and edge, 100 permutations and 100 bootstrap scores were then generated, and the method-specific *P*-value scores were merged using Brown’s method. False positives were detected and removed from the final network by applying Benjamini–Hochberg correction. Unstable edges with a score outside the 95% confidence interval, as defined by the bootstrap distribution, were discarded. Only edges supported by at least two methods and with *P*-values < .05 were conserved in the final network.

## Results

### Environmental conditions

Average air temperature and the number of days above freezing decreased with higher latitude (Supplementary Table S1). Correspondingly, water temperatures were highest in the lowest latitude water bodies from Kuujjuarapik (14.8–21°C) and Umiujaq (14.6–18.2°C) and lowest in the ice-based meltwater ponds of the ice shelves (0.4–2.8°C) (Table 1; Supplementary Table S2). On average, pH was lowest in the High Arctic ice shelf meltwater ponds (6.14–6.98) and highest in ponds and lakes around Cambridge Bay (8.16–9.84). Water conductivity varied from 29.5 to 1365 µS cm^−1^, with the lowest average values in Umiujaq (29.5–112.5 µS cm^−1^) and highest in Cambridge Bay (300–1365 µS cm^−1^).

### 16S rRNA and 18S rRNA gene diversity of cyanobacterial mat communities across the Canadian Arctic

**Figure 1.**
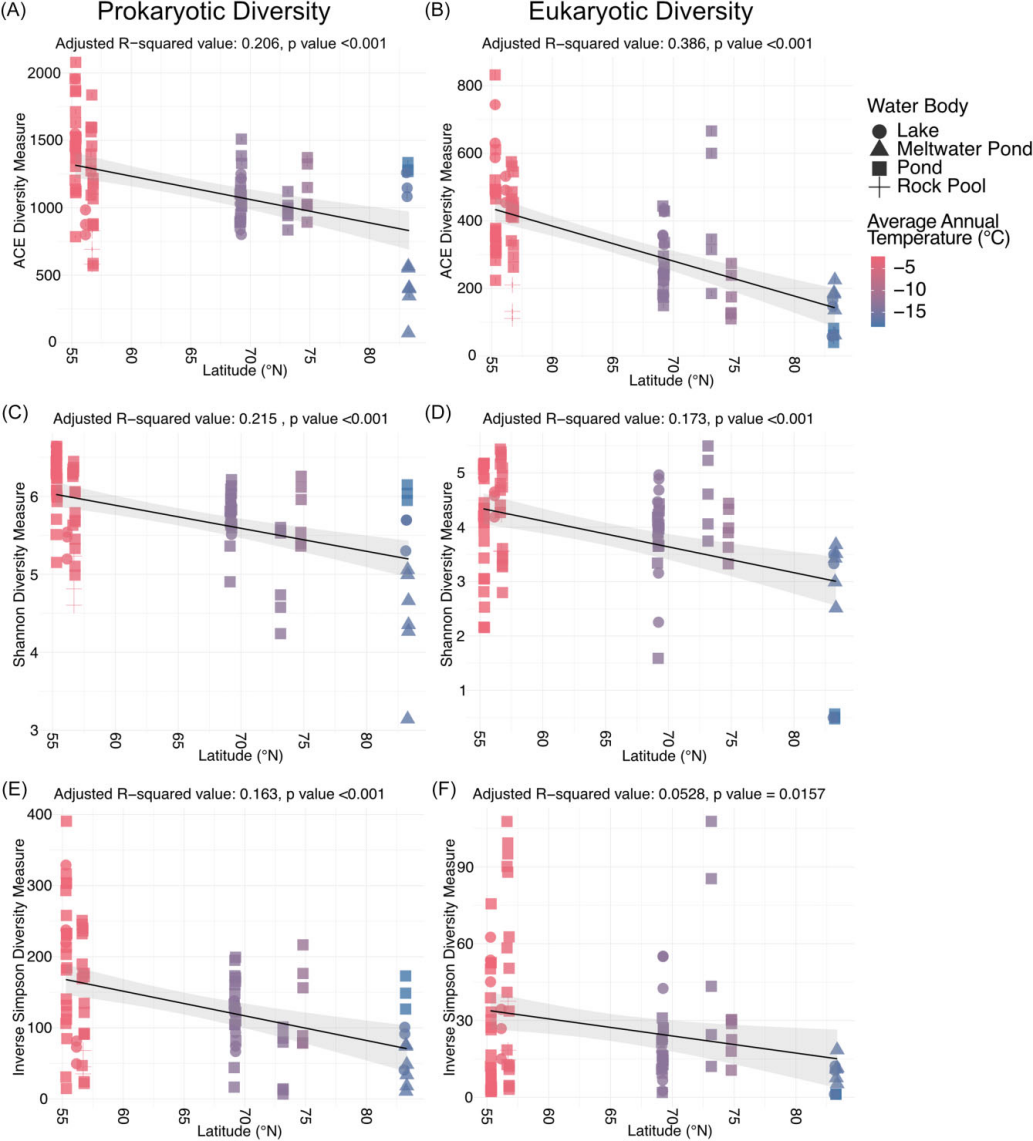
Alpha-diversity estimates for each sample plotted by latitude (55–83°N). Left column: prokaryotic diversity; right column: eukaryotic diversity. (A, B) ACE diversity index, (C, D) Shannon diversity index, and (E, F) inverse Simpson diversity index. A linear model was used to create a trend line based on the diversity index values plotted against latitudinal coordinates. Shaded area around the line represents the 95% confidence level interval.

Across all cyanobacterial mat communities, 16S rRNA gene richness (1148) and evenness (Shannon: 5.74, inverse Simpson: 134.47) were on average higher than 18S rRNA gene richness (337) and evenness (Shannon: 3.90, inverse Simpson: 27.59) (Supplementary File 1). A significant negative association between latitude and 16S rRNA and 18S rRNA gene richness (ACE diversity index, *P* < .001) was determined by linear regression (Fig. 1A and B). Shannon (*P* < .001) and inverse Simpson (*P* < .001) diversity indices of the 16S rRNA gene communities were also significantly negatively associated with latitude (Fig. 1C and E). The 18S rRNA gene communities were significantly correlated with latitude by the Shannon diversity index (*P* < .001) but not by inverse Simpson (*P* = .016) (Fig. 1D and F).

**Figure 2.**
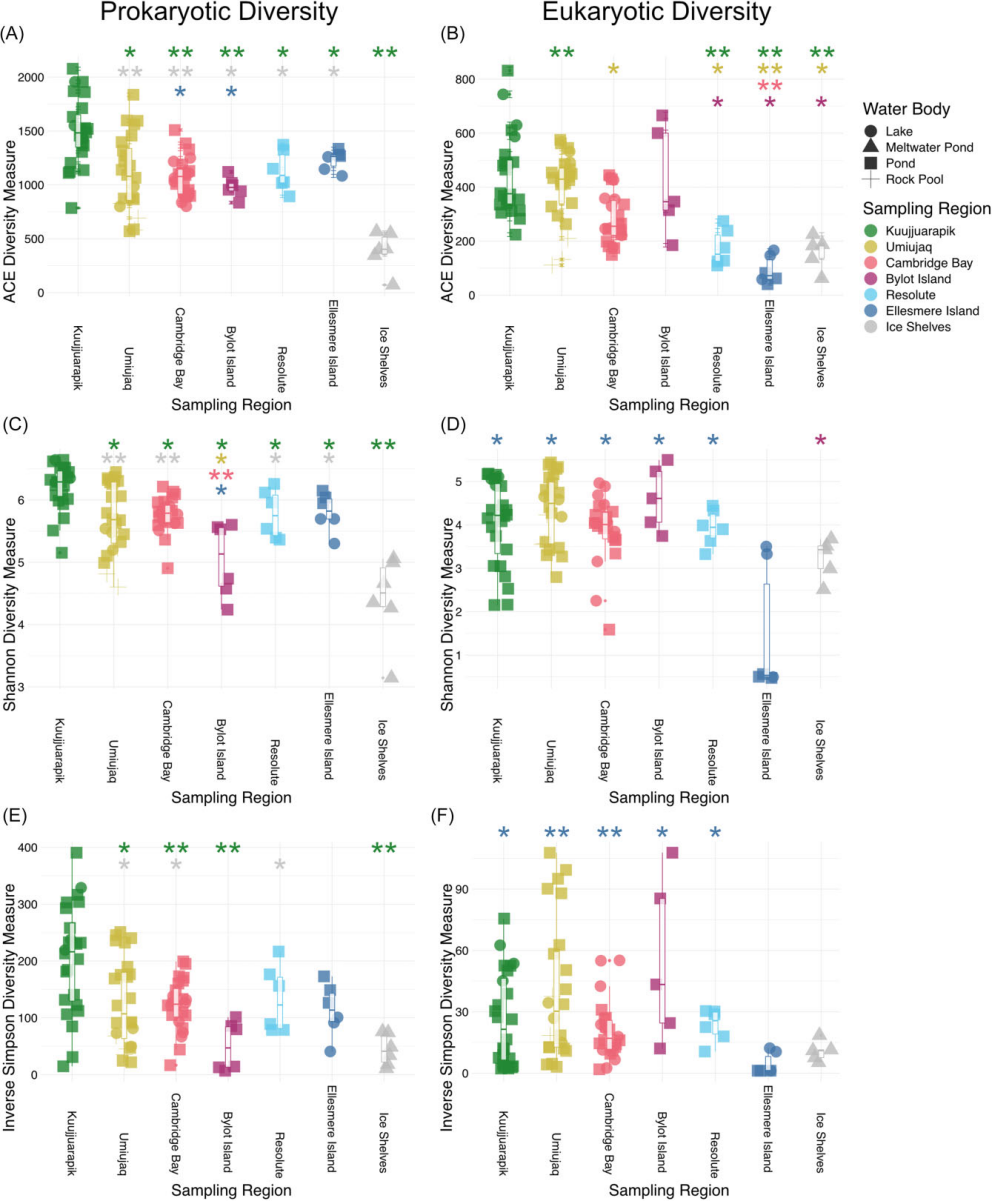
Alpha-diversity estimates for each sample grouped by sampling region, plotted left to right by latitude. Left column: prokaryotic diversity; right column: eukaryotic diversity. (A) and (B) = ACE diversity index, (C) and (D) = Shannon diversity index, and (E) and (F) = inverse Simpson diversity index. The boxplot represents the interquartile range with the median denoted by the bold central line. The whiskers represent 1.5 times the interquartile range and all outliers are denoted by a dot. Significant differences between sampling regions are denoted by an asterisk (*P-value < .01, **P-value < .001).

The mats from the lowest latitude sampling sites in Kuujjuarpik contained the highest mean average prokaryotic richness (1494.75) and evenness (Shannon = 6.22, inverse Simpson = 203.02) (Supplementary  File 1), significantly greater than that found in all higher latitude sampling sites by Wilcoxon signed-rank testing of ACE and Shannon diversity indices (Fig. 2; Supplementary File 1). The mats from the meltwater ponds of the High Arctic ice shelves had the lowest average richness (389.33) and evenness (Shannon = 4.41, inverse Simpson = 43.56) and had significantly lower diversity than all lower latitude sampling sites by ACE and Shannon diversity indices (Fig. 2; Supplementary File 1). Eukaryotic richness (ACE) was significantly higher in the Kuujjuarapik, Umiujaq, and Bylot Island sampling sites than all of the higher latitude sampling sites. Eukaryotic evenness (Shannon and inverse Simpson) was significantly lower in the Ellesmere Island (Antoniades Pond and Ward Hunt Lake) mat communities, compared to all lower latitude sampling regions.

### Abiotic drivers of cyanobacterial mat diversity

**Figure 3.**
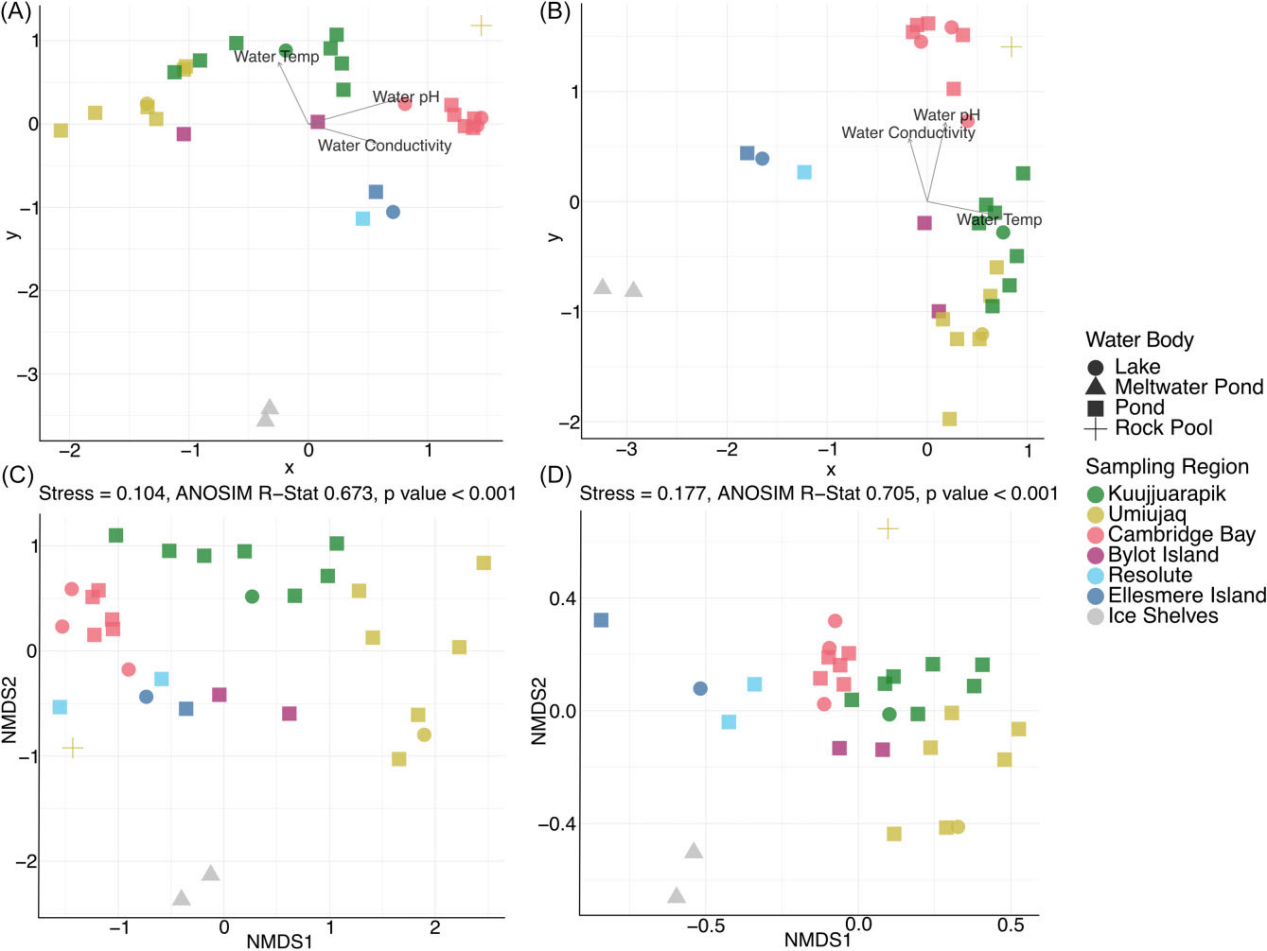
Canonical-correlation analysis of the (A) prokaryotic and (B) eukaryotic communities in the mat samples plotted along with sample metadata significantly associated with the ASV distribution between water bodies as determined by permutational multivariate analysis of variance (PERMANOVA). Sample data mapped on graph, length, and direction of arrow demonstrate strength of association with the communities. Ordination of the relative abundance count data of the (C) prokaryotic and (D) eukaryotic communites, averaged for each water body, by two-dimensional Bray–Curtis NMDS. Significant differences in Bray–Curtis distance measures were identified between sampling regions by analysis of similarities (ANOSIM).

The reduction in 16S rRNA and 18S rRNA gene richness and evenness in the cyanobacterial mat communities correlated with decreasing average annual temperatures across the latitudinal gradient (Fig. 1). Furthermore, richness and evenness (Shannon) of the mat communities significantly positively correlated with warmer water temperatures (*P* < .001) (Supplementary  Fig. S2). 16S rRNA and 18S rRNA gene composition in cyanobacterial mats from Kujjuarapik and Umiujaq sampling sites were most closely affiliated to the water temperature vector compared to all other sampling sites, by CCA (Fig. 3A and B). Cyanobacterial mats from Cambridge Bay were correlated with water conductivity and pH vectors. The mat communities from Resolute, Ellesmere Island, and the High Arctic ice shelves showed little association with the measured environmental variables.

Environmental influences on diversity were further assessed by Mantel tests (Supplementary File 1). Both 16S rRNA and 18S rRNA gene community composition positively correlated with average annual air temperatures (*r* = 0.214, *P* = .0017 and *r* = 0.117, *P* = .031, respectively). The 18S rRNA gene community also positively correlated with pH (*r* = 0.091, *P* = .046), which was not the case for the 16S rRNA gene community (*r* = 0.121, *P* = .070). Finally, a significant association between geographic proximity (Haversine distance) and community composition (Bray–Curtis dissimilarity) was identified in both 16S gene rRNA (*r* = 0.208, *P* = .0022) and 18S rRNA gene (*r* = 0.110, *P* = .040) composition across all sampled communities.

### Taxonomic composition of cyanobacterial mats

A significant difference in prokaryotic (*R*: 0.673, *P* < .0001) and eukaryotic (*R*: 0.705, *P* < .0001) community composition was identified between sampling sites (Fig. 3C and D). Overall cyanobacterial mat communities clustered by sampling sites, except for an overlap in the prokaryotic communities of Resolute and Ellesmere Island. The meltwater pond communities from the ice shelves were the most dissimilar from all other sampling regions. Eukaryotic communities from Kuujjuarapik, Umiujaq, Cambridge Bay, and Bylot Island were more similar to each other than Resolute, Ellesmere Island, and the ice shelves (Fig. 3D).

**Figure 4.**
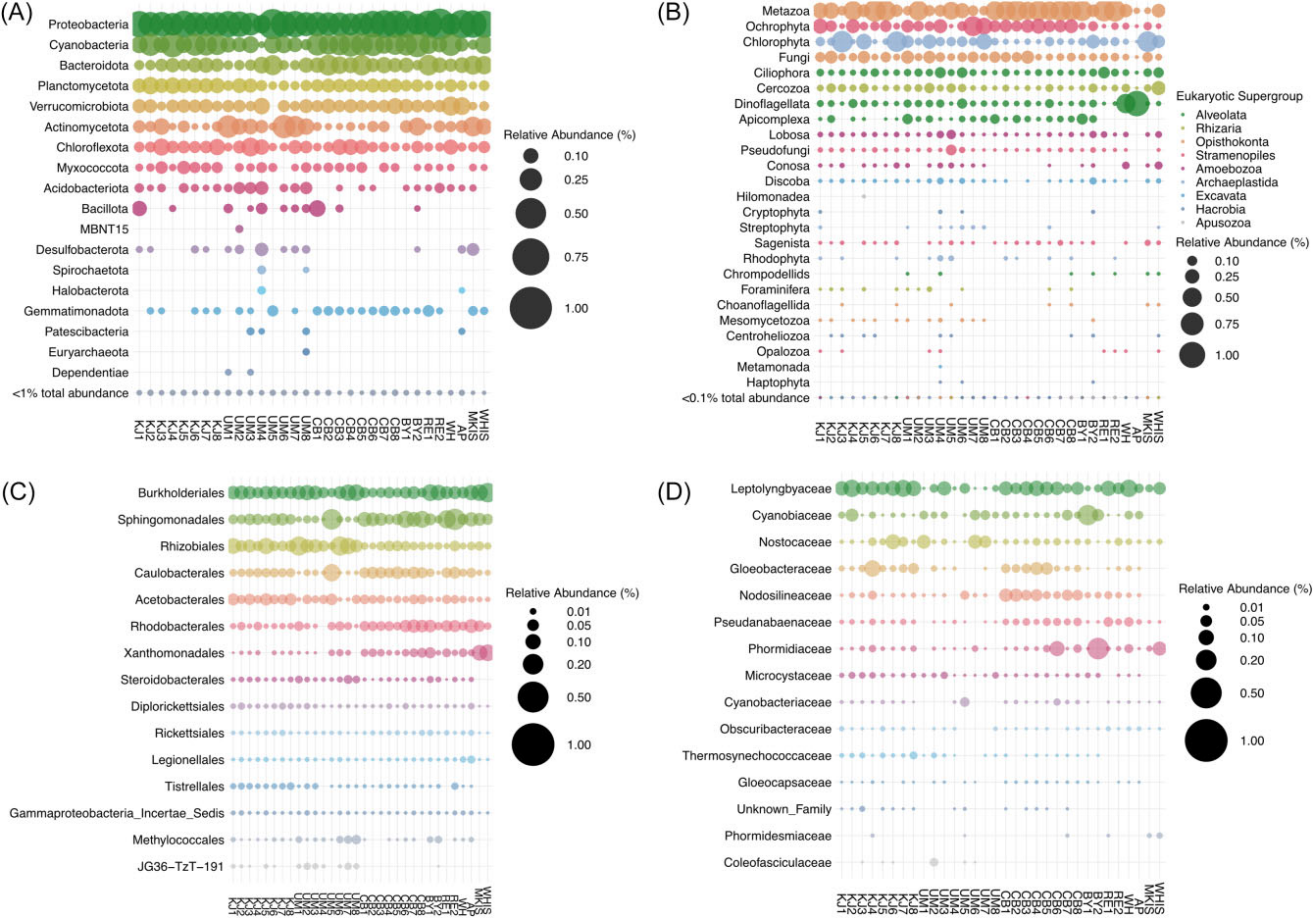
Taxonomic diversity of the microbial communities. The size of the bubble is determined by the relative abundance of that taxon within the sample. Samples are plotted by latitude from lowest to highest (55–83°N). (A) Prokaryotic phyla and (B) eukaryotic divisions, low abundance phyla, here defined as <1% of the total abundance for the prokaryotic community and 0.1% for the eukaryotic community, were grouped. The 15 most abundant families within (C) Proteobacteria and (D) Cyanobacteria. KJ = Kuujjuarapik, UM = Umiujaq, CB = Cambridge Bay, BY = Bylot Island, RE = Resolute, WH = Ward Hunt Lake, AP = Antoniades Pond, MKIS = Markham Ice Shelf, and WHIS = Ward Hunt Ice Shelf.

Across all cyanobacterial mat communities, 49 prokaryotic phyla were identified. The bacterial phyla: Proteobacteria (33%), Cyanobacteria (17%), Bacteroidota (10%), Actinomycetota (8%), Verrucomicrobiota (7%), Planctomycetota (7%), and Chloroflexota (6%), accounted for 88% of 16S rRNA gene ASVs and were present in all mat communities (Fig. 4A). Within the Proteobacteria, the Gammaproteobacteria order Burkholderiales (7%) and the Alphaproteobacteria orders Sphingomonadales (6%), Rhizobiales (5%), Caulobacterales (3%), Rhodobacterales (3%), and Acetobacterales (2%), were identified in all mat communities (Fig. 4C). The meltwater pond mat communities of the ice shelves had a greater proportion of proteobacteria ASVs (58%) than cyanobacterial mats from all other sampling sites. This included a higher proportion of Xanthomonadales (11%), which accounted for less than 3% of ASVs in all other sampling sites. On average, the most abundant cyanobacterial families were the *Leptolyngbyaceae* (6%), *Cyanobiaceae* (2%), *Phormidiaceae* (2%), and *Nostocaceae* (2%) (Fig. 4D). The meltwater ponds had the greatest abundance of *Phormidium* taxa.

Metazoa (36%), Ochrophyta (14%), Chlorophyta (14%), Fungi (9%), Dinoflagellata (8%), Cercozoa (5%), and Ciliophora (5%) accounted for 91% of 18S rRNA gene ASVs and were identified in all cyanobacterial mat communities (Fig. 4B). Metazoa identified included nematodes (Chromadorea and Haliplectoidea), platyhelminths, insects, crustacea, annelids, rotifers, and tardigrades. Crustacea (mostly copepods, ostracods, and fairy shrimp) and insects (mostly midge species, *Acricotopus lucens* and *Chironomus tepperi*) were proportionally more abundant in taiga (Kuujjuarapik and Umiujaq) and tundra (Cambridge Bay and Bylot Island) than polar desert (Resolute and Ellesmere Island) and meltwater pond (Ward Hunt and Markham ice shelves) cyanobacterial mat communities (Supplementary  Fig. S3). The meltwater ponds only contained rotifers and tardigrades. Taxonomic annotation of fungi was limited, with 37% of ASVs assigned at the class level (Supplementary Fig. S4). The most abundant fungal classes were the Ascomycota and Chytridiomycota. Microsporidiomycotina and Harpellales were present in taiga and tundra mat communities but were absent from higher latitude communities.

### Assessment of trophic functional diversity of protists from 18S rRNA gene metabarcoding

Functional annotation was possible for 4263 out of 6296 of all protist ASVs (68%), the rest were left unassigned (Supplementary File 2). Diverse phototrophic, mixotrophic, and heterotrophic taxonomic groups were identified in all cyanobacterial mat communities.

**Figure 5.**
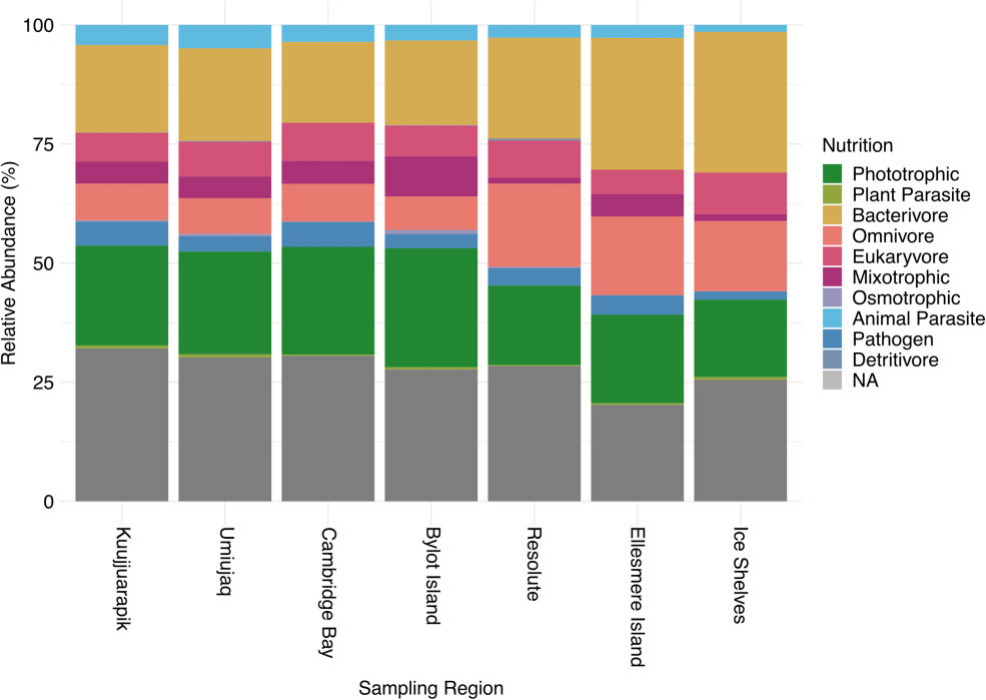
Relative abundance of ASVs assigned to each protist functional groups, averaged by sample regions, ordered from left to right by increasing latitude.

The relative abundance of phototrophs was greater in cyanobacterial mat communities between Kuujjuarapik and Bylot Island (21%–25%), than between Resolute and the High Arctic ice shelf samples (16%–19%) (Fig. 5). In contrast, bacterivore ASVs had proportionally higher abundance in the Ellesmere Island (28%) and High Arctic ice shelf samples (30%), than all lower latitude mat communities (17%–21%). Omnivore ASVs were also proportionally higher abundance between Resolute and the ice shelves (15%–18%) than all lower latitude sampling sites (7%–8%) (Fig. 5).

The phototrophic groups with the highest relative abundance were the Chlorophyta (green algae), Bacillariophyta (Diatoms), and Dinophyceae (Dinoflagellates) (Supplementary  Fig. S5A). Chrysophyceae were the most prevalent mixotrophic group, with further mixotrophic Gymnodiales (Dinoflagellates), Cryptomonadales (Cryptophyta), and Euglenophyceae (Discoba) identified in low relative abundance in mat communities between Kuujjuarapik and Bylot Island (Supplementary Fig. S5B).

Bacterivores, including grazers, filter feeders, and predators, were identified in the Lobosa and Conosa (Amoebozoa), the Cercozoa, Labyrinthulomycetes, Heterolobosea, and Euglenida (Supplementary  Fig. S5C). The most abundant eukaryvores were the Vampyrellida (Cercozoa); the Nassophorea, Litostomatea, Colpodea, and Suctoria (Ciliophora); and the Colpodellida (Apicomplexa) (Supplementary  Fig. S5D). Vampyrellida were identified in all mat communities except from Ward Hunt Ice Shelf. The most abundant omnivores were Ciliophora and Cercozoa (Supplementary  Fig. S5E).

Animal parasites were markedly less abundant in the High Arctic cyanobacterial mat communities (Supplementary Fig. S5F). The Apicomplexan orders Neogregarinorida and Eugregarinorida, including *Actinocephalidae, Monocystis*, and *Leidyana* spp., were absent from Ward Hunt lake, Antoniades Pond, and the Markham and Ward Hunt ice shelves. Hymenostomatia (Ciliophora), Icthyosponida (Mesomycetozoa), and all other parasitic lineages were identified in mat communities at all sampling sites except the ice shelves. The Cyrtophoria, Trypanosomatida, and Capsasporida were the only parasitic ASVs identified in mats from Ward Hunt and Markham Ice Shelf. The pathogenic oomycetes, Saprolegnieales, were also present in mats across all sampling sites (Fig. 5).

### Co-occurrence network analyses

**Figure 6.**
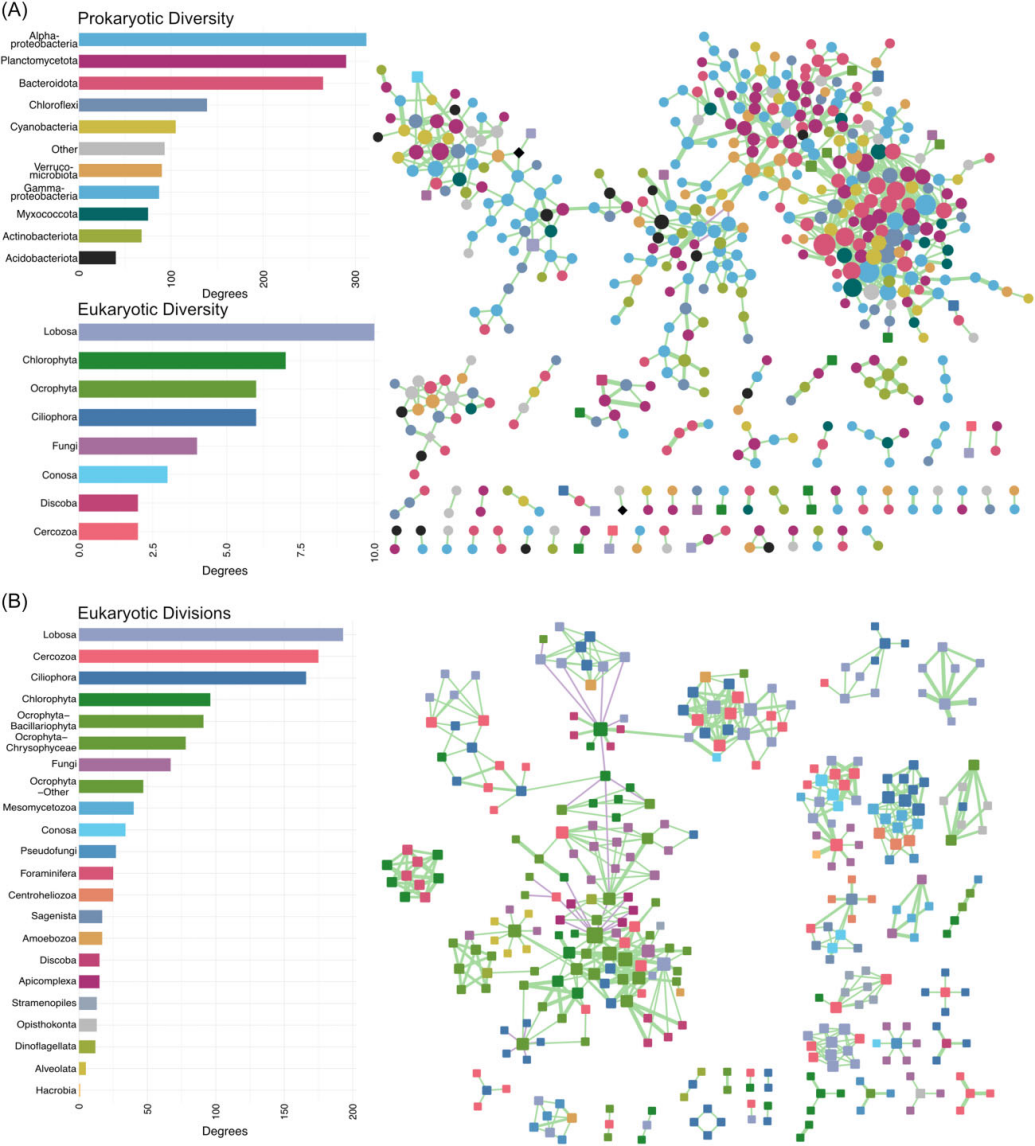
Co-occurrence network analysis calculated on the ASVs across all mat samples. The nodes represent a unique ASV, and the size of the nodes is proportional to the connection degree. The size of the edges are proportional to the number of methods that support the associated connection (two to five methods). The shape of the nodes indicates the domain: Bacteria (circles), Eukaryota (squares), and Archaea (diamonds). The barplots summarize the node degree distribution (number of connections) of each taxonomic group in the network. The green line indicates a positive connection and the pink a negative connection. (A) Prokaryotic protist interaction co-occurence network. (B) The eukaryotic protist interaction co-occurence network.

The prokaryotic protist interaction network contained 510 nodes (480 bacteria, 2 archaea, 24 protists, and 4 fungi) and 802 edges (Supplementary Table S3). The eukaryotic protist interaction network contained 340 nodes (309 protists, 31 fungi, and 6 unassigned Opisthokonta) and 586 edges (Supplementary Table S3). The structure of the two networks differed. The prokaryotic protist interaction network consisted of three joined subnetworks, 23 major hubnodes (containing >10 degrees), and several minor networks (21 nodes or fewer) (Fig. 6A). The eukaryotic network consisted of several smaller subnetworks and contained more negative interactions than the prokaryotic protist interaction network (50 to 8 ) (Fig. 6B). The eukaryotic network had a higher average number of neighbours (edge connections per node), a proxy for greater connectivity. The eukaryotic network had a higher proportion of edges produced from three to five significant methods and a higher average number of methods per edge than the combined network (2.51 to 2.3) (Supplementary Table S3).

Alphaproteobacteria, Planctomycetota, and Bacteroidota were the dominant prokaryotic orders in the prokaryotic protist interaction network. The major hubnodes were *Hyphomonadaceae* (Alphaproteobacteria), *Lewinella* (Bacteroidota), and *Anaerolineae* (Chloroflexi spp.). *Leptolyngbyaceae* accounted for 17 out of 32 cyanobacteria nodes. Eukaryotic groups were in low abundance (≤10 degrees) (Fig. 6A). Heterotrophic Lobosa, Ciliophora, and Cercozoa contributed the most interactions in the eukaryotic protist interaction network, including Vampyrellida and Colpodea (Fig. 6B). The major hubnodes were *Navicula* and *Nitzschia* spp. (Bacillariophyta).

## Discussion

### Arctic cyanobacterial mat diversity decreased across a latitudinal gradient

Across the Canadian Arctic, latitudinal gradients of decreasing temperature and light availability restrict biodiversity in the higher latitudes (Vincent 2010). The changes in terrestrial fauna and flora are classified into Arctic ecozones, from the boreal taiga, through tundra, to the polar desert (Wrona et al. 2016). These empirical classifications are an example of the macroecological theory of the latitude diversity gradient (LDG); diversity decreases with proximity to the polar regions (Pianka 1966). Previous studies in the Canadian Arctic have identified LDGs in freshwater macroinvertebrates (Lento et al. 2022) and marine microeukaryotes (Xu et al. 2022). To our knowledge, our study presents the first evidence of a LDG in cyanobacterial mats, microbial communities identified almost ubiquitously across freshwater systems in the Canadian Arctic (Jungblut et al. 2010, Vincent 2010). A significant negative regression was identified between prokaryotic and eukaryotic richness and evenness in cyanobacterial mats collected here from 55°N to 83°N.

The reduction in prokaryotic and eukaryotic richness with increasing latitude may be due to increasingly restrictive environmental conditions for primary production in the High Arctic regions (Jungblut et al. 2017). The composition of the taiga region mat communities correlated with warmer air and water temperatures. In high-latitude regions, colder temperatures correspond with extended periods of ice cover, low light, and winter darkness, reducing annual primary production (Charvet et al. 2014). Across our sampling sites, the average number of months above freezing ranged from one to six, from the highest to lowest latitude sampling regions. The lower annual daylight and prolonged ice-cover limit the energy supply to phototrophs, reducing primary production and biomass growth in the High Arctic mat communities (Jungblut et al. 2017, Vincent et al. 2000). The ‘species–energy hypothesis’ of the LDG states that reduced solar energy at higher latitudes reduces primary productivity, thus limiting species richness (Currie et al. 2004). Colder temperatures and fewer days with light availability are likely factors restricting organic carbon accumulation within higher latitude microbial mat communities, resulting in reduced diversity.

Diversity of ecological communities is shaped by dispersal dynamics, environmental conditions, and biotic interactions (Leibold et al. 2004). Mat community composition was consistently more similar within sampling sites than between, and the number of shared ASVs and community composition both increased with geographical proximity. This is consistent with previous biogeographical studies of microbial communities in the Canadian Arctic (Harding et al. 2011, Jungblut et al. 2012, Comte et al. 2018). Prokaryotic composition and taxonomic diversity were, however, more consistent across the LDG than eukaryotic communities within the cyanobacterial mats. This suggests that particular bacterial phyla are essential for mat formation and structure (Jungblut et al. 2010). Comparatively, eukaryotic richness and community composition in the taiga and tundra ecozones were more distinct from the High Arctic polar desert and ice shelf mat communities. The sparser structure of the eukaryotic network in comparison to the dense interactions in the prokaryotic community further supported greater heterogeneity in eukaryotic diversity across the Arctic ecozones. This is consistent with previous clone library surveys of Arctic mat communities (Jungblut et al. 2012). Finally, a significant association between pH and eukaryotic composition identified by the Mantel test was not identified in the prokaryotic communities. The distinctions in eukaryotic community composition across gradients of changing climatic conditions suggests that microbial eukaryotic diversity may be more sensitive to environmental factors than prokaryotic diversity. In keeping with sensitivity to external conditions, distinct eukaryotic communities were identified between annual winter and summer conditions in Arctic lakes (Bock et al. 2014) and ponds (Simon et al. 2015, Potvin et al. 2022).

Due to logistical constraints, our analysis did not include nutrient conditions. In particular, nitrogen and phosphorus concentrations would influence microbial growth and taxonomic diversity within cyanobacterial mats in polar regions (Varin et al. 2010, Velázquez et al. 2017). While our study encompassed a variety of freshwater ecosystem types in the Canadian High Arctic, it is possible that the relatively fewer sampling sites at higher latitudes could have led to an under-representation of richness in Canadian High Arctic communities. Future work integrating nutrient measurements and spanning a broader range of sites across the Canadian High Arctic or extending into regions like Alaska or Greenland would enhance our findings and further test the latitudinal gradient hypothesis.

### Cyanobacteria and heterotrophic bacteria and nutrient cycling in Arctic cyanobacterial communities

Microbial mats have been described as ‘jungles of biodiversity’ (Battin et al. 2016) and the organisms within them span the tree of life (Varin et al. 2010, Jungblut et al. 2012). Within all cyanobacterial mat communities, we identified a high diversity of bacteria, metazoa, fungi, and protists, as in previous studies in the Canadian Arctic (Jungblut et al. 2012, Mohit et al. 2017). Despite an overall reduction in richness with latitude, all mat communities were consistently dominated by Proteobacteria, Cyanobacteria, Bacteroidota, Actinobacteriota, Verrucomicrobiota, Planctomycetota, and Chloroflexota, as reported in previous 16S rRNA gene surveys of microbial mats from the polar regions (Kleinteich et al. 2017, Greco et al. 2020, Jackson et al. 2021).

Within these microbial jungles, the keystone species are the cyanobacteria (Jungblut et al. 2010). *Leptolyngbyaceae* and *Nostocaceae* were the most abundant cyanobacterial families in our communities, consistent with previous molecular and microscopy work on polar cyanobacterial mats (Vincent et al. 2000, Jungblut et al. 2010, Velázquez et al. 2017). *Leptolyngbyaceae* were also the most abundant cyanobacterial group in the prokaryotic protist interaction network, demonstrating their significance in both primary production and three-dimensional organization of polar microbial mat communities, providing a substrate for more specialized organisms (de los Ríos et al. 2004). The High Arctic mat communities had a higher proportion of *Phormidiaceae*, particularly *Tychonema. Phormidium* species may occur as a selective structural component to optimize carbon accumulation in low dissolved inorganic carbon environments, as discussed for Antarctic mat communities (Hawes et al. 2019). *Tychonema* spp. were identified as the most abundant cyanobacteria in pinnacle communities from Lake Untersee (Greco et al. 2020) and meltwater ponds in the McMurdo Ice Shelf (Jackson et al. 2021), in keeping with the similarity between High Arctic ice shelf meltwater pond cyanobacterial mat communities and Antarctic microbial communities (Jungblut et al. 2017, Kleinteich et al. 2017).

Proteobacteria, primarily from Alphaproteobacteria and Gammaproteobacteria, were the most abundant phyla in the mat communities, a consistent feature of Arctic (Varin et al. 2010, Harding et al. 2011), Antarctic (Kleinteich et al. 2017, Greco et al. 2020), and stream biofilms (Battin et al. 2016). Proteobacteria play an important role in decomposition and nutrient cycling in the mat communities. Several Alphaproteobacteria orders degrade humic substances, a major component of dissolved organic matter in freshwater environments (Battin et al. 2016). Varin et al. (2010) previously identified the high abundance of nitrogen cycling-related genes originating from Proteobacteria. The significance of these heterotrophic prokaryotes was further highlighted in the network analyses, with Alphaproteobacteria, Planctomycetota, Bacteroidota, and Chloroflexota forming the main hubnodes. This suggests nutrient turnover by heterotrophic bacteria is essential to mat community function, especially in the subsurface layers (Battin et al. 2016, Velázquez et al. 2017).

Planctomycetes were the second most connected prokaryotic phylum in the combined network. The contribution of planctomycetes to Arctic mat communities has been less discussed than cyanobacteria and proteobacteria; however, they have been described in Antarctic lake communities (Greco et al. 2020) and are commonly reported in biofilm communities from extreme oligotrophic environments, where they degrade algal biomass (Wiegand et al. 2018). Anaerobic planctomycete species are capable of anaerobic ammonium oxidation (anammox) and have been identified in aquatic biofilm communities (Lago and Bondoso 2014). Their contribution to the co-occurrence network suggests that planctomycetes may be key to degradation and nitrogen cycling in Arctic cyanobacterial mats.

### Protists increase the trophic complexity of Arctic cyanobacterial mat communities

The functional diversity of protists identified in freshwater environments has changed our understanding of trophic interactions in microbial food webs, where varied phototrophic and heterotrophic groups may occupy several trophic levels (Arndt and Nomdedeu 2016, Burki et al. 2021). Our 18S rRNA gene survey identified phototrophic and heterotrophic bacterivores, omnivores, and eukaryvores in all Arctic mat communities. This is consistent with the notion that protists are common contributors to trophic interactions within the ubiquitous benthic cyanobacterial mat communities found in terrestrial aquatic systems across the polar regions (Vincent et al. 2000, Jungblut et al. 2012). Within the eukaryotic network, a diversity of interactions between phototrophic, heterotrophic, and parasitic groups was identified, supporting their contribution to trophic interactions and nutrient transfer within mat communities. Furthermore, the greater number of negative associations in the eukaryotic network suggests that specific niches may be occupied by multiple taxa between different mat communities.

We identified a proportionally greater richness of phototrophic organisms in the taiga and tundra mat communities and a proportionally greater richness of bacterivores and omnivores in the High Arctic mat communities. These shifts in functional diversity may explain the distinction in eukaryotic composition of the cyanobacterial mats between these Arctic ecozones. The greater diversity of phototrophic organisms in the lower latitude regions is likely a response to the extended summer period in the lower latitude environments. This further highlights the influence of environmental conditions on eukaryotic diversity within the mat communities.

Phototrophic taxa had the highest relative abundance in the protist communities of the cyanobacterial mats, as identified in a previous global study of freshwater protist diversity (Singer et al. 2021). This is likely a reflection of the summer sampling period, during the months of maximum light availability. Phototrophic protists most likely dwell on the surface layer of the mats, attenuating the penetration of light into deeper layers of the cyanobacterial mats, permitting growth of subsurface heterotrophic communities and dim-light-adapted cyanobacteria (Vincent et al. 1993, Battin et al. 2003). The most abundant phototrophs in our mat communities, the Chlorophyta, reach maximum growth during the early summer in Antarctic mat systems (Velázquez et al. 2017). It is possible that their high relative abundance in our mat communities is a similar response to greater light availability. Mixotrophy is a common adaptive strategy in planktonic communities in High Arctic (Charvet et al. 2014) and perennially ice-covered Antarctic lakes (Bielewicz et al. 2011); however, mixotrophy was not a common trophic strategy within our mat communities. Mixotrophy is more energetically costly than heterotrophy, and therefore may be less favourable in more nutrient-rich environments (Charvet et al. 2014). Mixotrophy was, however, likely underestimated in our survey, with Dinoflagellata and Chlorophyta possibly having more mixotrophic representatives (Adl et al. 2019, Pang et al. 2021).

Across the latitudinal gradient, Cercozoa, Ciliophora, and Lobosa were the most abundant heterotrophic protists identified in the mat communities and in the co-occurrence networks, consistent with a global metabarcoding survey of soil and freshwater environments (Singer et al. 2021). Cercozoa were mostly from the Filosa, which display gliding mobility; the microbial mats would provide a suitable substrate for such grazing heterotrophic activity (Bass et al. 2009). Vampyrellida, the most abundant eukaryvores in the mat communities, are specialized predators of filamentous algae and cyanobacteria, and are common to freshwaters, soils (Hess and Suthaus 2022), and polar cryoconites (Millar et al. 2021). Vampyrellida exhibit fast population growth in response to algal blooms (Hess and Suthaus 2022). Predatory Vampyrellida may therefore act as regulators of bloom cycles in the abundant phototrophic algae and cyanobacteria within the mat communities during the summer growth period.

Ciliophora are common contributors to microbial mat communities (Dopheide et al. 2008, Battin et al. 2016). We identified a high abundance in all Arctic mat communities, comprising heterotrophic bacterivores, eukaryvores, and parasites. Ciliates were previously identified as heterotrophic dominants in the winter community of a river ecosystem in southern Canada (Cruaud et al. 2019), and important members of the winter microbiome in a subarctic river (Blais et al. 2024). As such, they may play a key role in nutrient recycling in the Arctic mat communities during the winter months, in habitats that do not freeze to the bottom. Lobosa were the most abundant bacterivores in our mat communities and are known grazers of microbial mats (Tekle et al. 2022).

The most striking shift in protist diversity across the Arctic ecozones was the reduction in animal parasites in the High Arctic cyanobacterial mats, suggesting a significant biogeographical barrier, most likely the restriction on host organisms, as identified within the metazoa in this study. Of the animal parasites identified, the most abundant were the Apicomplexa, diverse parasites of arthropods and crustacea (del Campo et al. 2019); the fish parasites, Hymenostomatia (Ciliophora) and Icthyosponida (Mesomycetozoa) (Adl et al. 2019); and the Harpellales, symbiotic fungi of insects (Wang et al. 2016). All of these groups were absent from the Markham and Ward Hunt ice shelves. These findings suggest that parasites could be used to gauge the northward reductions of ecological barriers under climate change that are influenced by complex biotic and abiotic factors (Kutz et al. 2014). However, it is possible that certain phylogenetic groups were undetected due to PCR biases. Protists span an enormous diversity across the eukaryotic domain, making it difficult to design universal primers that cover all taxonomic groups (Burki et al. 2021). PCR-free shotgun sequencing or targeted PCR approaches are needed to convincingly test for the presence of animal parasitic protists in cyanobacterial mats in High Arctic freshwater environments.

## Conclusions

Cyanobacterial mats are important for biomass and productivity in Arctic freshwater environments. This study showed that prokaryotic and microbial eukaryotic species richness in freshwater microbial mats decreases along a latitudinal gradient from the subarctic to High Arctic based on 16S rRNA and 18S rRNA gene sequencing. Cyanobacteria and Proteobacteria were consistently the most abundant phototrophic and heterotrophic bacterial contributors to microbial mats, respectively. We identified a diversity of microbial eukaryotes comprising various physiologies and potentially contributing to many trophic interactions within abundant microbial mat communities across the Canadian Arctic, increasing phototrophic input during the summer months and facilitating nutrient turnover through diverse heterotrophic roles. Changes in eukaryotic diversity were distinct across latitudinal gradients from the subarctic to High Arctic, including a notable increase in phototrophic microbial eukaryotes in lower latitude taiga and tundra communities and an increased relative abundance of heterotrophic protists in High Arctic communities. Furthermore, the reduction in eukaryotic richness included a reduction in metazoa and potential parasites of higher organisms in the High Arctic. The identified responsiveness of protists to changing environmental conditions across these northern latitudes of Canada highlights their contribution to biodiversity, and their importance in understanding and monitoring biodiversity change in the Arctic.

## Supplementary Material

fiae067_Supplemental_Files
