# Supplementary material for: Arctic cyanobacterial mat community diversity decreases with latitude across the Canadian Arctic
Source: FEMS Microbiol Ecol. 2024 Apr 23;100(6):fiae067. doi: 10.1093/femsec/fiae067 (PMC11092279; doi:10.1093/femsec/fiae067)
Supplement: fiae067_Supplemental_Files [file fiae067_supplemental_files.zip › Figure Legends for Supplementary Figures.docx]

**Figure Legends for Supplementary Figures**

**Supplementary Figure 1.** Microbial mats were sampled from water bodies proximal to research stations in Kuujjuarapik (55°) and Umiujaq (56°) in the taiga ecozone, Cambridge Bay (69°), Bylot Island (73°), and Resolute (74°) within the tundra ecozone, and the High Arctic mat communities were sampled from Antoniades’ Pond, Ellesmere Island (83°) and Ward Hunt Lake, Ward Hunt Island (83°), and from meltwater ponds on the Ward Hunt and Markham Ice Shelves (83°).

**Supplementary Figure 2.** Alpha diversity estimates for each sample plotted against water temperature. Left column = prokaryotic diversity, right column = eukaryotic diversity. A-B = ACE diversity index, C-D = Shannon diversity index, E-F = Inverse Simpson diversity index. A linear model was used to create a trend line based on the diversity index values plotted against latitudinal coordinates. Shaded area around the black line represents the 95% confidence level interval.

**Supplementary Figure 3.** The fifteen highest relative abundance orders of Metazoa identified by 18S rRNA gene metabarcoding across the microbial mat communities. KJ = Kuujjuarapik, UM = Umiujaq, CB = Cambridge Bay, BY = Bylot Island, RE = Resolute, WH = Ward Hunt Lake, AP = Antoniades Pond, MKIS = Markham Ice Shelf, WHIS = Ward Hunt Ice Shelf.

**Supplementary Figure 4.** The fourteen highest relative abundance orders of Fungi identified by 18S rRNA gene metabarcoding across the microbial mat communities. KJ = Kuujjuarapik, UM = Umiujaq, CB = Cambridge Bay, BY = Bylot Island, RE = Resolute, WH = Ward Hunt Lake, AP = Antoniades Pond, MKIS = Markham Ice Shelf, WHIS = Ward Hunt Ice Shelf.

**Supplementary File 5.** Fifteen taxa with the highest relative abundance within each protist functional group, (A) phototrophic, (B) mixotrophic, (C) bacterivores, (D), eukaryvores, (E) omnivores, and (F) animal parasites. KJ = Kuujjuarapik, UM = Umiujaq, CB = Cambridge Bay, BY = Bylot Island, RE = Resolute, WH = Ward Hunt Lake, AP = Antoniades Pond, MKIS = Markham Ice Shelf, WHIS = Ward Hunt Ice Shelf.
