# Supplementary material for: Arctic cyanobacterial mat community diversity decreases with latitude across the Canadian Arctic
Source: FEMS Microbiol Ecol. 2024 Apr 23;100(6):fiae067. doi: 10.1093/femsec/fiae067 (PMC11092279; doi:10.1093/femsec/fiae067)
Supplement: fiae067_Supplemental_Files [file fiae067_supplemental_files.zip › Supplementary Data_Table_1.docx]

**Supplementary Table 1.** Climate data for Canadian Arctic sampling sites. Climate stations chosen were nearest to sampling sites and historical monthly temperature was averaged for each station. All temperatures given in °C.

| **Region** | **Station Name** | **Jan** | **Feb** | **Mar** | **Apr** | **May** | **Jun** | **Jul** | **Aug** | **Sep** | **Oct** | **Nov** | **Dec** | **Ave Ann**  **Temp** | **Data Source** | **DOI** |
| --- | --- | --- | --- | --- | --- | --- | --- | --- | --- | --- | --- | --- | --- | --- | --- | --- |
| Kuujjuarapik | KJRAPIK | -20.02 | -21.22 | -14.94 | -5.28 | 2.84 | 8.92 | 12.03 | 12.81 | 8.79 | 3.95 | -2.74 | -12.05 | -2.24 | Nordicana D | DOI: 10.5885/45057SL-EADE4434146946A7 |
| Umiujaq | UMIROCA | -20.44 | -21.37 | -15.38 | -6.69 | 1.96 | 7.76 | 11.60 | 11.88 | 7.75 | 2.90 | -4.08 | -11.64 | -2.98 | Nordicana D | DOI: 10.5885/45120SL-067305A53E914AF0 |
| Cambridge Bay | Cambridge Bay A | -30.71 | -31.13 | -29.61 | -20.84 | -9.82 | 1.90 | 8.83 | 7.58 | 1.24 | -8.38 | -20.60 | -27.20 | -13.23 | Canadian Government Climate Archives | Climate ID: 2400600 |
| Bylot Island | BYLOSIL | -31.52 | -31.84 | -28.53 | -18.28 | -6.01 | 2.84 | 6.64 | 5.49 | -0.45 | -8.25 | -20.27 | -27.94 | -13.18 | Nordicana D | DOI: 10.5885/45039SL-EE76C1BDAADC4890 |
| Resolute Bay | Resolute CARS | -27.56 | -28.44 | -25.56 | -17.47 | -7.31 | 2.78 | 7.74 | 5.13 | -1.58 | -9.05 | -18.19 | -22.88 | -11.87 | Canadian Government Climate Archives | Climate ID: 2403500 |
| Ward Hunt Island | ELLWARH | -29.77 | -31.35 | -31.99 | -21.49 | -10.01 | -0.50 | 1.59 | -0.51 | -7.87 | -17.95 | -24.29 | -27.45 | -16.80 | Nordicana D | DOI: 10.5885/44985SL-8F203FD3ACCD4138 |
| Antoniades Pond | ELLLACA | -31.88 | -33.68 | -34.33 | -24.71 | -10.06 | 0.03 | 1.82 | -0.02 | -8.48 | -19.86 | -27.12 | -31.41 | -18.31 | Nordicana D | DOI: 10.5885/44985SL-8F203FD3ACCD4138 |
