# Supplementary material for: Arctic cyanobacterial mat community diversity decreases with latitude across the Canadian Arctic
Source: FEMS Microbiol Ecol. 2024 Apr 23;100(6):fiae067. doi: 10.1093/femsec/fiae067 (PMC11092279; doi:10.1093/femsec/fiae067)
Supplement: fiae067_Supplemental_Files [file fiae067_supplemental_files.zip › Supplementary Data_Table_2.docx]

**Supplementary Table 2.** Sample metadata for cyanobacterial mats. KJ = Kuujjuarapik, UM = Umiujaq, CB = Cambridge Bay, BY = Bylot Island, RE = Resolute, WH = Ward Hunt Lake, AP = Antoniades Pond, MKIS = Markham Ice Shelf, WHIS = Ward Hunt Ice Shelf.

| **Sample ID** | **Sample Location** | **Water Body** | **Arctic Ecozone** | **Latitude** | **Longitude** | **Sample Year** | **Temperature (°C)** | **pH** | **Conductivity (μS cm-1)** |
| --- | --- | --- | --- | --- | --- | --- | --- | --- | --- |
| KJ1.1 | Kuujjuarapik | Pond | Taiga | 55.283 | -77.739 | 2017 | 19.6 | 7.25 | 60 |
| KJ1.2 | Kuujjuarapik | Pond | Taiga | 55.283 | -77.739 | 2017 | 19.6 | 7.23 | 60 |
| KJ1.3 | Kuujjuarapik | Pond | Taiga | 55.283 | -77.739 | 2017 | 19.7 | 7.33 | 61 |
| KJ2.1 | Kuujjuarapik | Lake | Taiga | 55.284 | -77.737 | 2017 | 19.1 | 7.9 | 49 |
| KJ2.2 | Kuujjuarapik | Lake | Taiga | 55.287 | -77.736 | 2017 | 18.7 | 8.03 | 47 |
| KJ2.3 | Kuujjuarapik | Lake | Taiga | 55.287 | -77.737 | 2017 | 19.1 | 7.91 | 48 |
| KJ3.1 | Kuujjuarapik | Pond | Taiga | 55.314 | -77.734 | 2017 | 20.7 | 7.47 | 67 |
| KJ3.2 | Kuujjuarapik | Pond | Taiga | 55.314 | -77.735 | 2017 | 21.1 | 7.51 | 63 |
| KJ3.3 | Kuujjuarapik | Pond | Taiga | 55.314 | -77.735 | 2017 | 20.7 | 7.47 | 67 |
| KJ3.4 | Kuujjuarapik | Pond | Taiga | 55.314 | -77.735 | 2017 | 20.7 | 7.47 | 67 |
| KJ4.1 | Kuujjuarapik | Pond | Taiga | 55.323 | -77.715 | 2017 | 15.9 | 6.61 | 40 |
| KJ4.2 | Kuujjuarapik | Pond | Taiga | 55.323 | -77.715 | 2017 | 18 | 7.57 | 56 |
| KJ4.3 | Kuujjuarapik | Pond | Taiga | 55.323 | -77.715 | 2017 | 18.1 | 7.31 | 56 |
| KJ5.1 | Kuujjuarapik | Pond | Taiga | 55.326 | -77.717 | 2017 | 17 | 6.73 | 38 |
| KJ5.2 | Kuujjuarapik | Pond | Taiga | 55.326 | -77.717 | 2017 | 17 | 6.73 | 38 |
| KJ5.3 | Kuujjuarapik | Pond | Taiga | 55.326 | -77.717 | 2017 | 17 | 6.63 | 38 |
| KJ6.1 | Kuujjuarapik | Pond | Taiga | 55.333 | -77.696 | 2017 | 20.4 | 8.95 | 65 |
| KJ6.2 | Kuujjuarapik | Pond | Taiga | 55.333 | -77.696 | 2017 | 19.3 | 9.37 | 72 |
| KJ6.3 | Kuujjuarapik | Pond | Taiga | 55.333 | -77.698 | 2017 | 19.1 | 8.72 | 75 |
| KJ7.1 | Kuujjuarapik | Pond | Taiga | 55.334 | -77.697 | 2017 | 16.4 | 7.7 | 110 |
| KJ7.2 | Kuujjuarapik | Pond | Taiga | 55.334 | -77.697 | 2017 | 16.7 | 7.59 | 111 |
| KJ7.3 | Kuujjuarapik | Pond | Taiga | 55.334 | -77.697 | 2017 | 16.6 | 7.86 | 106 |
| KJ8.1 | Kuujjuarapik | Pond | Taiga | 55.361 | -77.651 | 2017 | 14.8 | 8.86 | 263 |
| KJ8.2 | Kuujjuarapik | Pond | Taiga | 55.361 | -77.651 | 2017 | 14.8 | 8.83 | 264 |
| KJ8.3 | Kuujjuarapik | Pond | Taiga | 55.361 | -77.651 | 2017 | 14.8 | 8.8 | 263 |
| UM1.1 | Umiujaq | Lake Inflow | Taiga | 56.155 | -76.308 | 2009 | 18.2 | 6.8 | 37 |
| UM1.2 | Umiujaq | Lake Inflow | Taiga | 56.155 | -76.308 | 2009 | 17.8 | 7.1 | 37 |
| UM1.3 | Umiujaq | Lake Inflow | Taiga | 56.155 | -76.308 | 2009 | 17.9 | 6.9 | 36.7 |
| UM2.1 | Umiujaq | Pond | Taiga | 56.620 | -76.626 | 2009 | 17.5 | 7.08 | 58.3 |
| UM2.2 | Umiujaq | Pond | Taiga | 56.620 | -76.626 | 2009 | 17.7 | 7.01 | 57.2 |
| UM2.3 | Umiujaq | Pond | Taiga | 56.620 | -76.626 | 2009 | 17.5 | 7.08 | 57.1 |
| UM3.1 | Umiujaq | Pond | Taiga | 56.622 | -76.601 | 2009 | 17.5 | 7.7 | 36.1 |
| UM3.2 | Umiujaq | Pond | Taiga | 56.622 | -76.601 | 2009 | 18 | 7.1 | 37.8 |
| UM3.3 | Umiujaq | Pond | Taiga | 56.622 | -76.601 | 2009 | 17.8 | 7.3 | 35.8 |
| UM4.1 | Umiujaq | Pond | Taiga | 56.682 | -76.707 | 2009 | 16.2 | 5.9 | 29.8 |
| UM4.2 | Umiujaq | Pond | Taiga | 56.682 | -76.707 | 2009 | 16.2 | 5.9 | 29.8 |
| UM4.3 | Umiujaq | Pond | Taiga | 56.682 | -76.707 | 2009 | 16.2 | 5.9 | 29.8 |
| UM5.1 | Umiujaq | Rock Pool | Taiga | 56.709 | -76.616 | 2009 | 15.5 | 9.66 | 112.5 |
| UM5.2 | Umiujaq | Rock Pool | Taiga | 56.709 | -76.616 | 2009 | 15.5 | 9.66 | 112.5 |
| UM5.3 | Umiujaq | Rock Pool | Taiga | 56.709 | -76.616 | 2009 | 15.5 | 9.66 | 112.5 |
| UM6.1 | Umiujaq | Pond | Taiga | 56.789 | -76.296 | 2009 | 14.6 | 6.7 | 36.8 |
| UM6.2 | Umiujaq | Pond | Taiga | 56.789 | -76.296 | 2009 | 14.7 | 7.15 | 36.7 |
| UM6.3 | Umiujaq | Pond | Taiga | 56.789 | -76.296 | 2009 | 15.7 | 6.83 | 36.4 |
| UM7.1 | Umiujaq | Pond | Taiga | 56.792 | -76.313 | 2009 | 15.2 | 7.1 | 29.7 |
| UM7.2 | Umiujaq | Pond | Taiga | 56.792 | -76.313 | 2009 | 15.9 | 6.79 | 30.1 |
| UM7.3 | Umiujaq | Pond | Taiga | 56.792 | -76.313 | 2009 | 15.6 | 6.75 | 29.5 |
| UM8.1 | Umiujaq | Pond | Taiga | 56.795 | -76.517 | 2009 | 16.7 | 6.4 | 32.6 |
| UM8.2 | Umiujaq | Pond | Taiga | 56.795 | -76.517 | 2009 | 17 | 6.3 | 31.2 |
| UM8.3 | Umiujaq | Pond | Taiga | 56.795 | -76.517 | 2009 | 16.7 | 6.4 | 32.6 |
| CB1.1 | Cambridge Bay | Pond | Tundra | 69.129 | -105.087 | 2017 | 17.3 | 9.4 | 758 |
| CB1.2 | Cambridge Bay | Pond | Tundra | 69.129 | -105.087 | 2017 | 17 | 9.37 | 759 |
| CB1.3 | Cambridge Bay | Pond | Tundra | 69.129 | -105.087 | 2017 | 17 | 9.31 | 761 |
| CB2.1 | Cambridge Bay | Lake | Tundra | 69.136 | -105.053 | 2017 | 8.8 | 8.92 | 376 |
| CB2.2 | Cambridge Bay | Lake | Tundra | 69.136 | -105.053 | 2017 | 8.8 | 8.84 | 376 |
| CB2.3 | Cambridge Bay | Lake | Tundra | 69.136 | -105.053 | 2017 | 8.9 | 8.79 | 374 |
| CB3.1 | Cambridge Bay | Pond | Tundra | 69.184 | -104.691 | 2017 | 10.2 | 8.99 | 990 |
| CB3.2 | Cambridge Bay | Pond | Tundra | 69.184 | -104.691 | 2017 | 10.3 | 9.04 | 992 |
| CB3.3 | Cambridge Bay | Pond | Tundra | 69.184 | -104.691 | 2017 | 10.1 | 9.06 | 993 |
| CB4.1 | Cambridge Bay | Pond | Tundra | 69.184 | -104.696 | 2017 | 10 | 9.84 | 763 |
| CB4.2 | Cambridge Bay | Pond | Tundra | 69.184 | -104.696 | 2017 | 10.2 | 8.92 | 763 |
| CB4.3 | Cambridge Bay | Pond | Tundra | 69.184 | -104.696 | 2017 | 10 | 8.84 | 767 |
| CB5.1 | Cambridge Bay | Lake | Tundra | 69.210 | -104.786 | 2017 | 15.5 | 8.18 | 1322 |
| CB5.2 | Cambridge Bay | Lake | Tundra | 69.210 | -104.787 | 2017 | 18.1 | 8.36 | 1284 |
| CB5.3 | Cambridge Bay | Lake | Tundra | 69.210 | -104.787 | 2017 | 18.2 | 8.16 | 1302 |
| CB6.1 | Cambridge Bay | Lake | Tundra | 69.243 | -104.760 | 2017 | 17.5 | 8.34 | 300 |
| CB6.2 | Cambridge Bay | Lake | Tundra | 69.243 | -104.760 | 2017 | 17.6 | 8.38 | 303 |
| CB6.3 | Cambridge Bay | Lake | Tundra | 69.243 | -104.760 | 2017 | 17.6 | 8.38 | 303 |
| CB7.1 | Cambridge Bay | Pond | Tundra | 69.243 | -104.760 | 2017 | 12.2 | 8.84 | 1365 |
| CB7.2 | Cambridge Bay | Pond | Tundra | 69.243 | -104.760 | 2017 | 11.7 | 8.9 | 1365 |
| CB7.3 | Cambridge Bay | Pond | Tundra | 69.243 | -104.760 | 2017 | 11.8 | 8.9 | 1361 |
| CB8.1 | Cambridge Bay | Pond | Tundra | 69.264 | -104.754 | 2017 | 15.5 | 8.49 | 552 |
| CB8.2 | Cambridge Bay | Pond | Tundra | 69.264 | -104.754 | 2017 | 15.1 | 8.42 | 552 |
| CB8.3 | Cambridge Bay | Pond | Tundra | 69.266 | -104.754 | 2017 | 15.5 | 8.44 | 549 |
| BY1.1 | Bylot Island | Pond | Tundra | 73.150 | -79.967 | 2009 | 13.3 | 7.55 | 100 |
| BY1.2 | Bylot Island | Pond | Tundra | 73.150 | -79.967 | 2009 | 13.3 | 7.55 | 100 |
| BY1.3 | Bylot Island | Pond | Tundra | 73.150 | -79.967 | 2009 | 13.3 | 7.55 | 100 |
| BY2.1 | Bylot Island | Pond | Tundra | 73.150 | -79.967 | 2009 | 15.3 | 6.59 | 79 |
| BY2.2 | Bylot Island | Pond | Tundra | 73.150 | -79.967 | 2009 | 15.3 | 6.59 | 79 |
| BY2.3 | Bylot Island | Pond | Tundra | 73.150 | -79.967 | 2009 | 15.3 | 6.59 | 79 |
| RE1.1 | Resolute Bay | Pond | Polar Desert | 74.763 | -95.212 | 2008 | NA | 8 | 560 |
| RE1.2 | Resolute Bay | Pond | Polar Desert | 74.763 | -95.212 | 2008 | NA | 8 | 560 |
| RE1.3 | Resolute Bay | Pond | Polar Desert | 74.763 | -95.212 | 2008 | NA | 8 | 560 |
| RE2.1 | Resolute Bay | Pond | Polar Desert | 74.790 | -95.090 | 2008 | 5.7 | 7.32 | 533 |
| RE2.2 | Resolute Bay | Pond | Polar Desert | 74.790 | -95.090 | 2008 | 7.3 | 7.34 | 500 |
| RE2.3 | Resolute Bay | Pond | Polar Desert | 74.790 | -95.090 | 2008 | 7.4 | 7.35 | 584 |
| WH1 | Ward Hunt Island | Lake | Polar Desert | 83.113 | -74.214 | 2007 | 1.8 | 7.45 | 83.7 |
| WH2 | Ward Hunt Island | Lake | Polar Desert | 83.164 | -74.180 | 2007 | 2.1 | 8.02 | 127 |
| WH3 | Ward Hunt Island | Lake | Polar Desert | 83.167 | -74.345 | 2007 | 1.6 | 8.15 | 129.2 |
| AP1 | Antoniades Pond | Pond | Polar Desert | 83.233 | -75.445 | 2007 | 6 | 8.28 | 137 |
| AP2 | Antoniades Pond | Pond | Polar Desert | 83.233 | -75.445 | 2007 | 6 | 8.28 | 137 |
| AP3 | Antoniades Pond | Pond | Polar Desert | 83.233 | -75.445 | 2007 | 6 | 8.28 | 137 |
| MKIS1 | Markham Ice Shelf | Meltwater Pond | Ice Shelf | 83.266 | -71.726 | 2007 | 1.1 | 6.52 | 492 |
| MKIS2 | Markham Ice Shelf | Meltwater Pond | Ice Shelf | 83.266 | -71.726 | 2007 | 2.8 | 6.79 | 779 |
| MKIS3 | Markham Ice Shelf | Meltwater Pond | Ice Shelf | 83.266 | -71.749 | 2007 | 1.4 | 6.29 | 640 |
| WHIS1 | Ward Hunt Ice Shelf | Meltwater Pond | Ice Shelf | 83.330 | -74.511 | 2007 | 0.8 | 6.98 | 364 |
| WHIS2 | Ward Hunt Ice Shelf | Meltwater Pond | Ice Shelf | 83.331 | -74.496 | 2007 | 1.5 | 6.24 | 740 |
| WHIS3 | Ward Hunt Ice Shelf | Meltwater Pond | Ice Shelf | 83.333 | -74.451 | 2007 | 0.4 | 6.14 | 50.3 |
