# Supplementary material for: Arctic cyanobacterial mat community diversity decreases with latitude across the Canadian Arctic
Source: FEMS Microbiol Ecol. 2024 Apr 23;100(6):fiae067. doi: 10.1093/femsec/fiae067 (PMC11092279; doi:10.1093/femsec/fiae067)
Supplement: fiae067_Supplemental_Files [file fiae067_supplemental_files.zip › Supplementary Data_Table_3.docx]

**Supplementary Table 3.** Summary statistics for the two co-occurrence networks. Nodes represent individual ASVs, Degree is the mean average number of neighbours for each node, and Edges are the number of connections between two nodes. Ed5-2 refer to the number of edges validated by 5-2 co-occurrence methods. Average method number is the average number of significant co-occurrence methods between two nodes across the total network. Average sample count is the average number of samples containing a particular node.

| **Network** | **Nodes** | **Degree** | **Edges** | **Ed5** | **Ed4** | **Ed3** | **Ed2** | **No. of negative degrees** | **No. of positive degrees** | | **Average method number** | **Average sample count** |
| --- | --- | --- | --- | --- | --- | --- | --- | --- | --- | --- | --- | --- |
| 16S rRNA gene, protist- and fungi-assigned ASVs | 510 | 3.14 | 802 | 3 | 36 | 160 | 603 | 8 | 1596 | 2.30 | | 12.3 |
| 18S ASVs only | 340 | 3.44 | 586 | 4 | 57 | 176 | 349 | 50 | 1122 | 2.51 | | 15.1 |
