# Supplementary figures and images for: Arctic cyanobacterial mat community diversity decreases with latitude across the Canadian Arctic
Source: FEMS Microbiol Ecol. 2024 Apr 23;100(6):fiae067. doi: 10.1093/femsec/fiae067 (PMC11092279; doi:10.1093/femsec/fiae067)

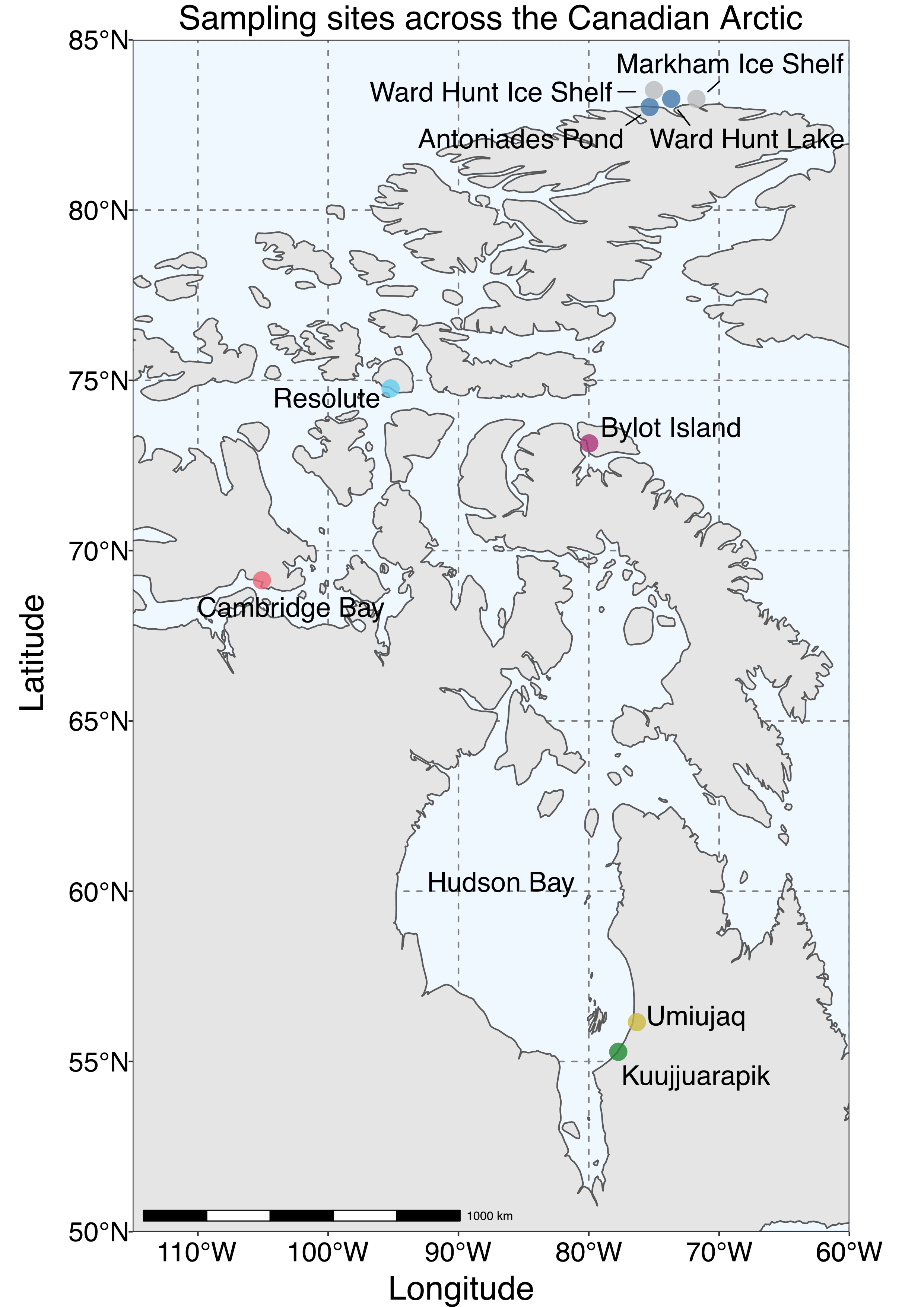

Supplement: fiae067_Supplemental_Files [file fiae067_supplemental_files.zip › Supplementary Data_Figure_1.tiff]

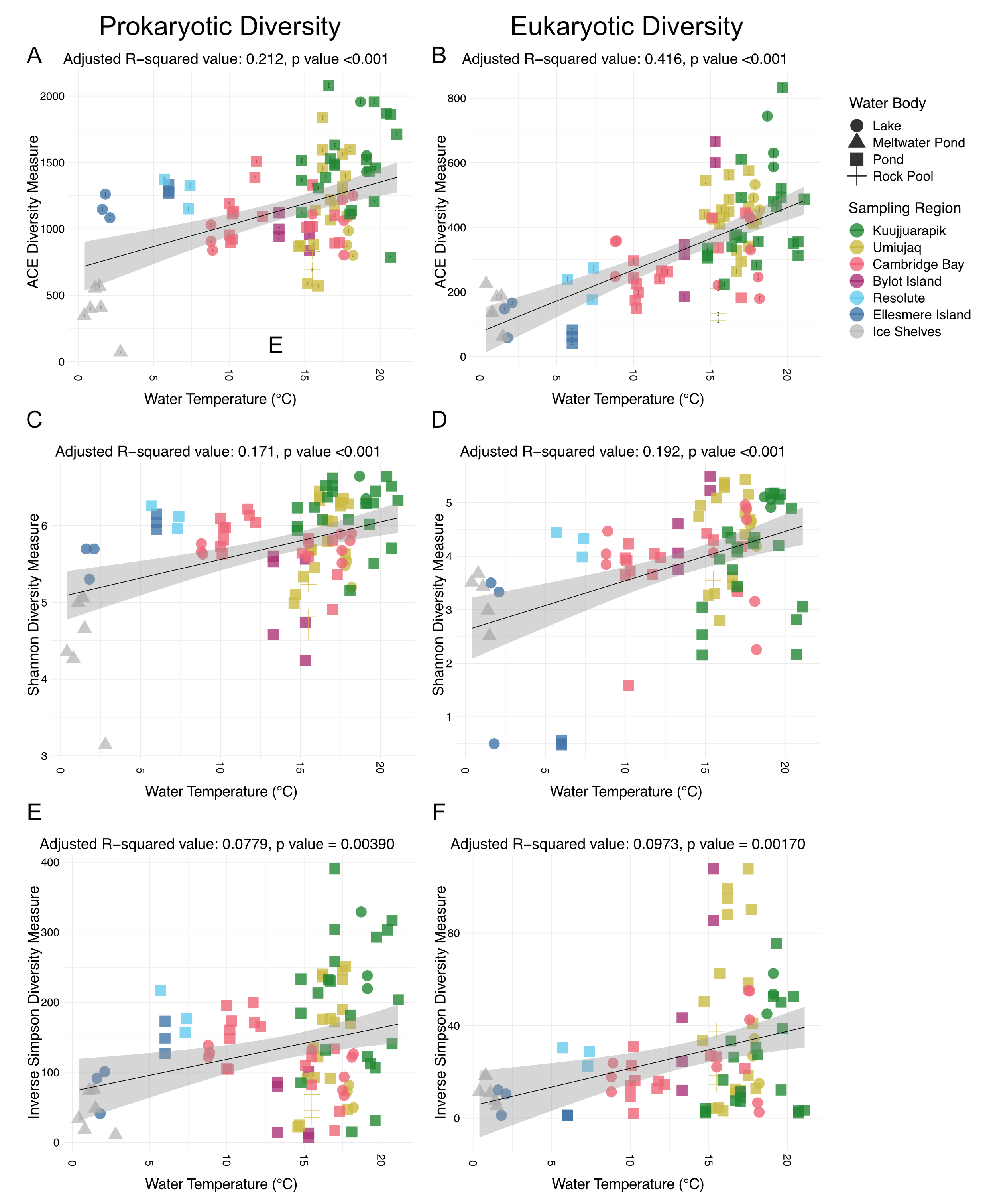

Supplement: fiae067_Supplemental_Files [file fiae067_supplemental_files.zip › Supplementary Data_Figure_2.tiff]

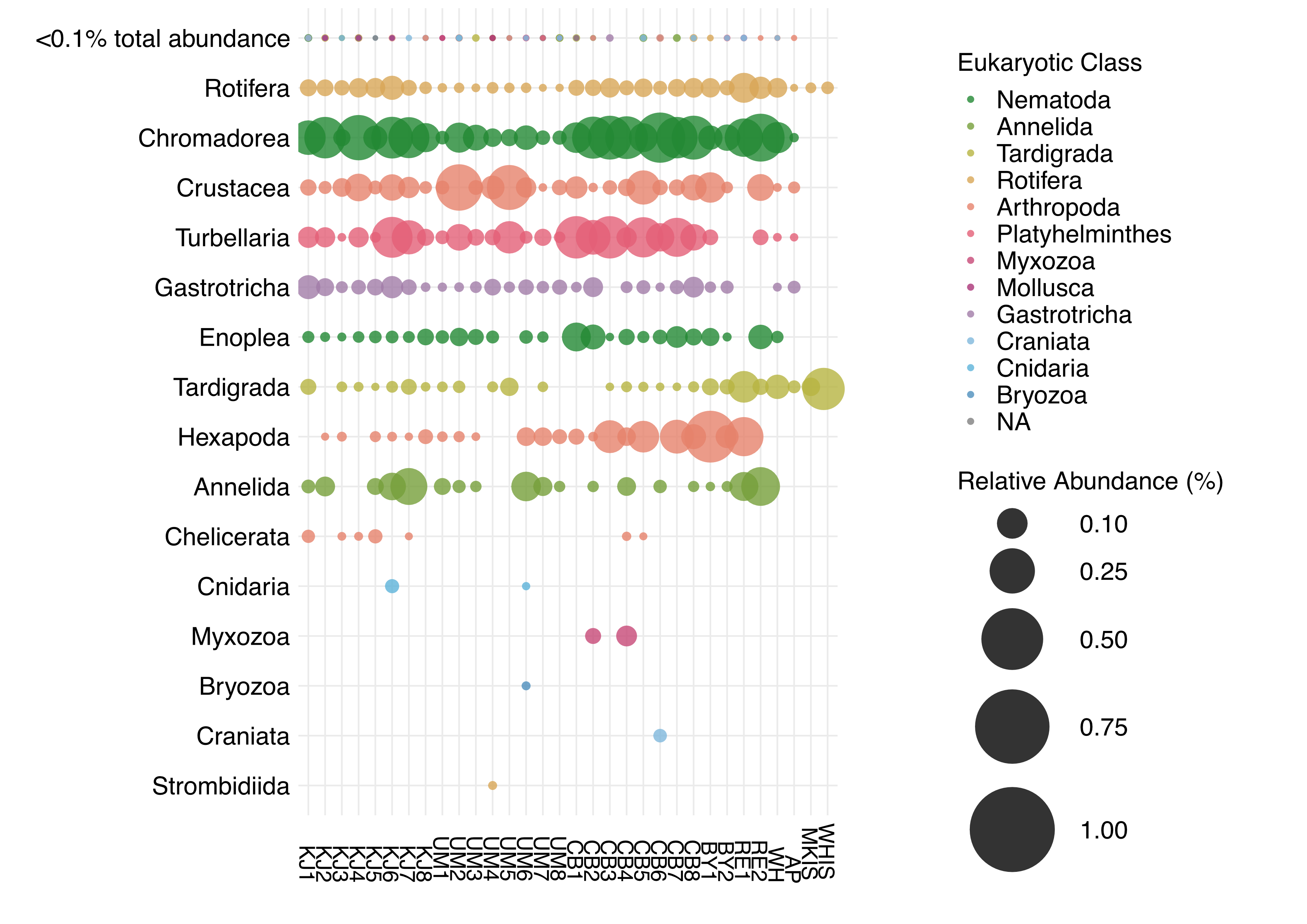

Supplement: fiae067_Supplemental_Files [file fiae067_supplemental_files.zip › Supplementary Data_Figure_3.tiff]

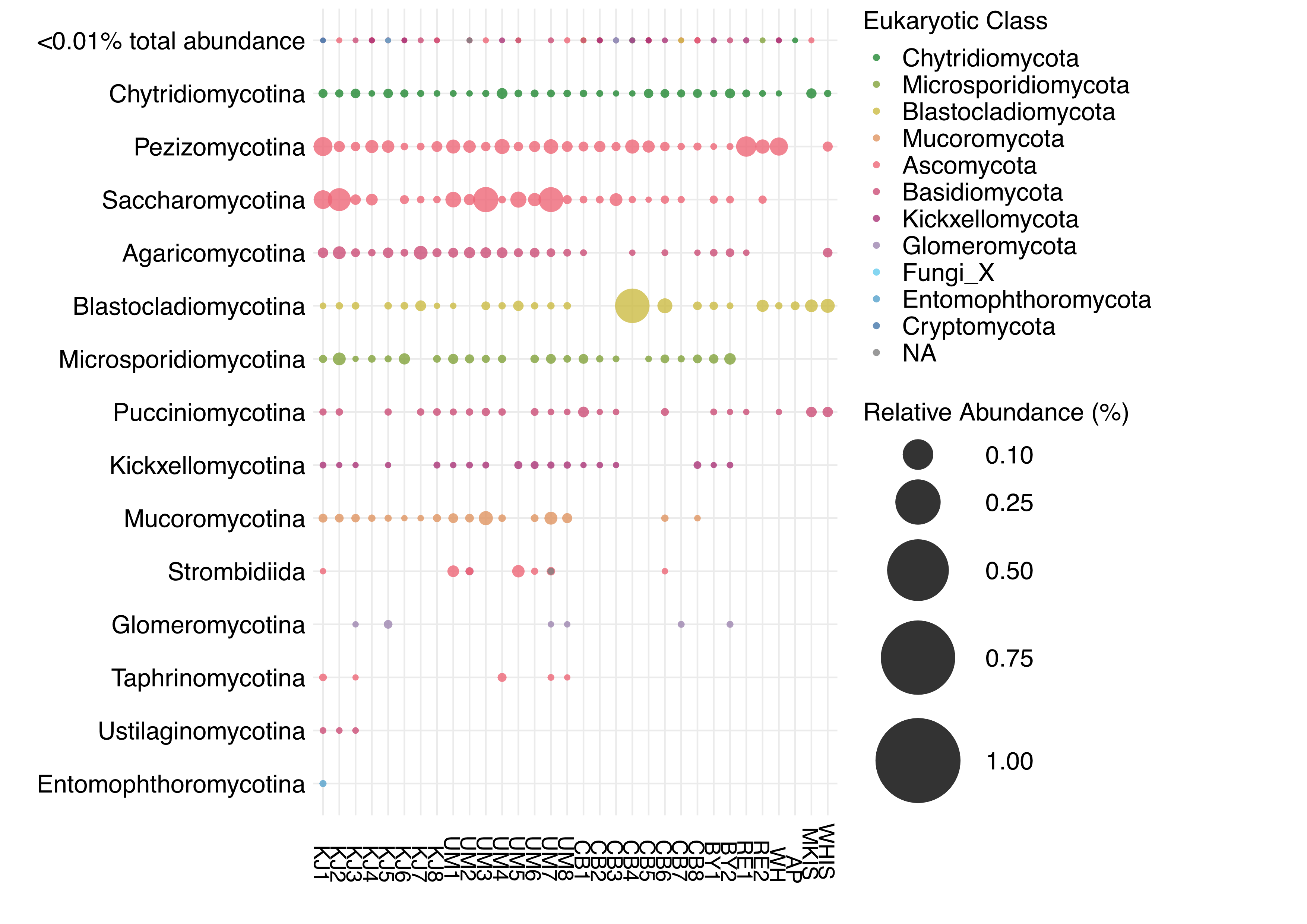

Supplement: fiae067_Supplemental_Files [file fiae067_supplemental_files.zip › Supplementary Data_Figure_4.tiff]

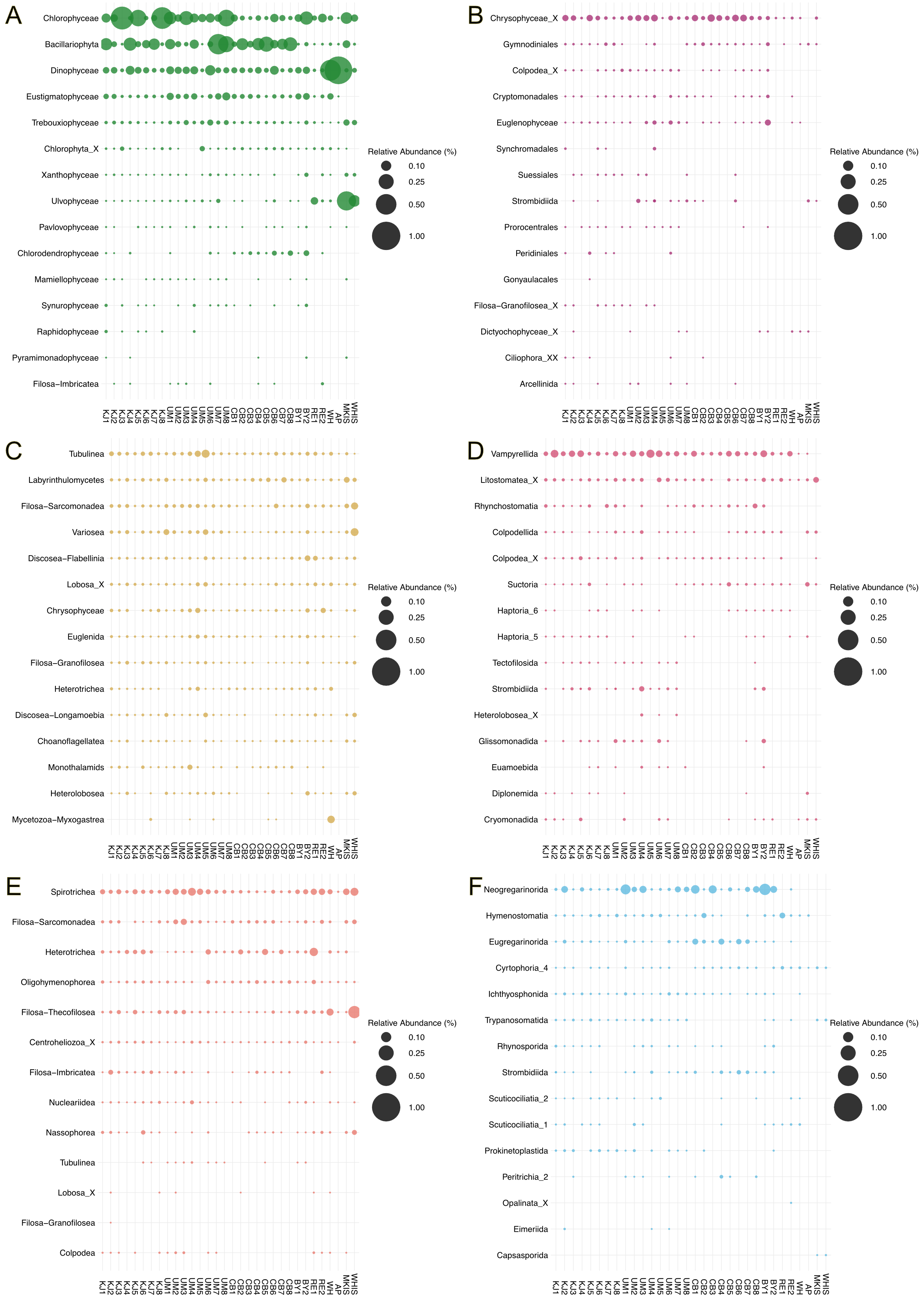

Supplement: fiae067_Supplemental_Files [file fiae067_supplemental_files.zip › Supplementary Data_Figure_5.tiff]
